# An investigation of broad-spectrum antibiotic-induced liver injury based on the FDA Adverse Event Reporting System and retrospective observational study

**DOI:** 10.1038/s41598-024-69279-6

**Published:** 2024-08-06

**Authors:** Chihiro Shiraishi, Hideo Kato, Toru Ogura, Takuya Iwamoto

**Affiliations:** 1 Department of Pharmacy, Mie University Hospital, Tsu, 514-8507 Japan; 2 Division of Clinical Medical Science, Department of Clinical Pharmaceutics, Mie University Graduate School of Medicine, Tsu, 514-8507 Japan; 3 Clinical Research Support Center, Mie University Hospital, Tsu, 514-8507 Japan

**Keywords:** Medical research, Risk factors

## Abstract

Tazobactam/piperacillin and meropenem are commonly used as an empiric treatment in patients with severe bacterial infections. However, few studies have investigated the cause of tazobactam/piperacillin- or meropenem-induced liver injury in them. Our objective was to evaluate the association between tazobactam/piperacillin or meropenem and liver injury in the intensive care unit patients. We evaluated the expression profiles of antibiotics-induced liver injury using the US Food and Drug Administration Adverse Event Reporting System (FAERS) database. Further, in the retrospective observational study, data of patients who initiated tazobactam/piperacillin or meropenem in the intensive care unit were extracted. In FAERS database, male, age, the fourth-generation cephalosporin, carbapenem, *β*-lactam and* β*-lactamase inhibitor combination, and complication of sepsis were associated with liver injury (*p* < 0.001). In the retrospective observational study, multivariate logistic regression analyses indicated that the risk factors for liver injury included male (*p* = 0.046), administration period ≥ 7 days (*p* < 0.001), and alanine aminotransferase (*p* = 0.031). Not only administration period but also sex and alanine aminotransferase should be considered when clinicians conduct the monitoring of liver function in the patients receiving tazobactam/piperacillin or meropenem.

## Introduction

Nearly 20% of patients receiving antibiotics for at least 24 h experience adverse events^1^. It is estimated that antibiotics are prescribed 10 times more frequently in intensive care unit (ICU) than in general wards^2^, thus significantly increasing the risk of adverse events in ICU patients, moreover, ICU patients face the risk of drug accumulation because of organ failure and sensitivity to drug reactions because of their unstable status, thus leading to diverse forms of toxicity including hepatotoxicity, neurological dysfunction, and acute kidney injury.

In ICU setting, the most common causes of hepatocellular injury are hypoxic hepatitis, congestive hepatopathy, septic shock, and drug-induced liver damage^3^. Antibiotics are the most common reason for drug induced liver injury (64%)^4^. Tazobactam/piperacillin (TZP), which is a combination of a *β*-lactam antibiotic and a *β*-lactamase inhibitor, and meropenem (MEM), which is a carbapenem, are commonly used as an empiric treatment in ICU patients with severe bacterial infections^5–7^ and are associated with a significantly increased incidence of liver injury^8–10^. However, the frequency of severe antibiotic-related hepatotoxicity is lower than that of antibiotic-related nephrotoxicity (liver injury vs. renal dysfunction: TZP, 5–6% vs. 9%; MEM, 4% vs. 21%)^11,12^. Therefore, it remains difficult to assess the cause of TZP- or MEM-induced hepatotoxicity. Typology of the injury can be classified as hepatitis (mostly due to hepatocyte necrosis), cholestatic (bile duct damage or cholangiolitis) or mixed cholestasis/hepatitis injury^13^. The liver injury associated with TZP or MEM is probably hypersensitivity or allergy^13^, while the cholestatic hepatitis is probably immunological-mediated tissue damage^14^. Previous study revealed that the occurrence of liver injury is not related the TZP or MEM dosage^15^, however, there is no data about the risk factors for TZP- or MEM-induced liver injury in ICU patients with serious infection.

First, we investigated the association between liver injury and clinical profiles in patients receiving broad-spectrum antibiotics including TZP or MEM by using the database of the US Food and Drug Administration Adverse Event Reporting System (FAERS). Finally, a retrospective observational study was conducted to evaluate the risk factors for TZP- or MEM-induced liver injury in ICU patients with serious infection. Given that our study was a single-center retrospective observational study, propensity score matching (PSM) analyses were conducted to reduce the bias of patient background. In addition, we conducted a sub-group (TZP or MEM) analysis to detect the independent risk factor for liver injury of TZP and MEM.

## Results

### FAERS database study

**Figure 1:**
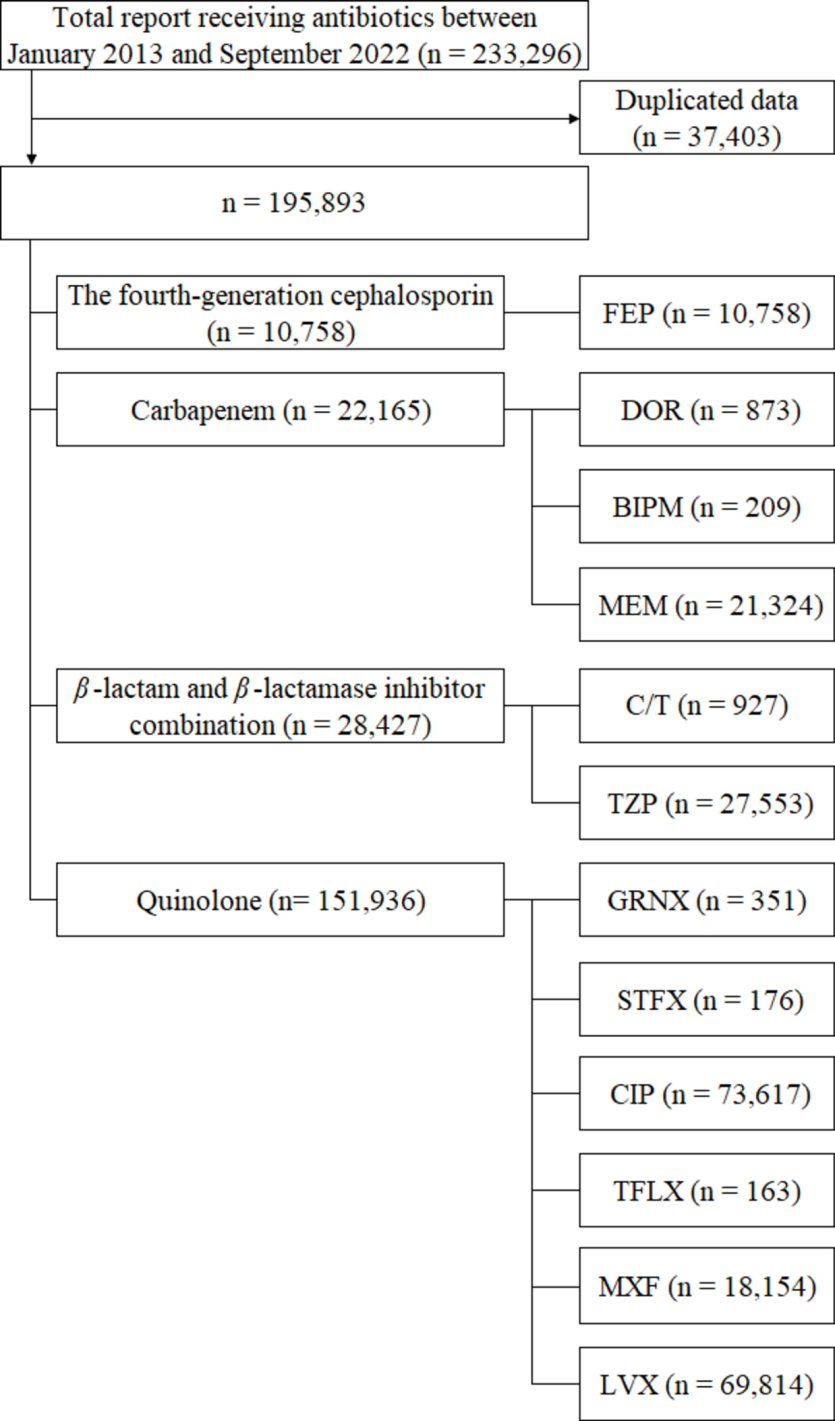
Flow chart of the process for data collection in the study using FAERS database. *BIPM* biapenem, *CIP* ciprofloxacin, *C/T* ceftolozane–tazobactam, *DOR* doripenem, *FAERS* the US Food and Drug Administration Adverse Event Reporting System, *FEP* cefepime, *GRNX* garenoxacin, *LVF* levofloxacin, *MEM* meropenem, *MXF* moxifloxacin, *STFX* sitafloxacin, *TFLX* tosufloxacin, *TZP* tazobactam/piperacillin. Multiple answers were allowed.

Total of 195,893 reports were extracted from the FAERS database. Figure 1 shows a flowchart of the patient. These reports include the fourth-generation cephalosporin (n = 10,758), carbapenem (n = 22,165), *β*-lactam and *β*-lactamase inhibitor combination (n = 28,427), and quinolone (n = 151,936). The number of reports attributed to the development of drug-induced liver injury was 1159 (11%). Table 1 shows the patient characteristics between the liver and non-liver injury groups. The reporting odds ratio (ROR) and 95% confidence interval (CI) for the above variables were as follows: male (ROR 1.237, 95% CI 1.090–1.405, *p* = 0.001), age (ROR 0.989, 95% CI 0.986–0.992, *p* < 0.001), the fourth–generation cephalosporin (ROR 1.400, 95% CI 1.124–1.743, *p* = 0.003), carbapenem (ROR 1.845, 95% CI 1.592–2.138, *p* < 0.001), *β*-lactam and *β*-lactamase inhibitor combination (ROR 1.383, 95% CI 1.194–1.603, *p* < 0.001), and complication of sepsis (ROR 1.705, 95% CI 1.126–2.582, *p* = 0.012).

**Table 1:** Demographic and clinical characteristics and risk analysis of liver injury in the study using FAERS database.

Variables	Liver injury (n = 1159)	Non-liver injury (n = 194,734)	ROR [95% CI]	*p*-value
	n (%)	n (%)		
Sex				
Female	465 (40.1)	93,521 (48.0)		
Male	494 (42.6)	80,296 (41.2)	1.237 [1.090–1.405]	0.001
Unknown	200 (17.3)	20,917 (10.7)		
Age*, years	54.0 [36.8–67.0]	61.0 [45.0–72.0]	0.989 [0.986–0.992]	< 0.001
Unknown	223 (19.2)	47,914 (24.6)		
The fourth-generation cephalosporin	87 (7.5)	10,671 (5.5)	1.400 [1.124–1.743]	0.003
FEP	87 (7.5)	10,671 (5.5)	1.400 [1.124–1.743]	0.003
Carbapenem	220 (19.0)	21,945 (11.3)	1.845 [1.592–2.138]	< 0.001
DOR	6 (0.5)	867 (0.4)	1.164 [0.520–2.603]	0.712
BIPM	10 (0.9)	199 (0.1)	8.508 [4.496–16.100]	< 0.001
MEM	208 (17.9)	21,116 (10.8)	1.798 [1.547–2.091]	< 0.001
*β*-Lactam and *β*-lactamase inhibitor combination	220 (19.0)	28,207 (14.5)	1.383 [1.194–1.603]	< 0.001
C/T	0 (0.0)	927 (0.5)	–	–
TZP	220 (19.0)	27,333 (14.0)	1.435 [1.238–1.663]	< 0.001
Quinolone	849 (73.3)	151,087 (77.6)	0.791 [0.694–0.901]	< 0.001
GRNX	6 (0.5)	345 (0.2)	2.932 [1.305–6.585]	0.009
STFX	5 (0.4)	171 (0.1)	4.930 [2.022–12.018]	< 0.001
CIP	335 (28.9)	73,282 (37.6)	0.674 [0.593–0.765]	< 0.001
TFLX	5 (0.4)	158 (0.1)	5.336 [2.186–13.021]	< 0.001
MXF	175 (15.1)	17,979 (9.2)	1.748 [1.488–2.055]	< 0.001
LVX	371 (32.0)	69,443 (35.7)	0.849 [0.751–0.961]	0.010
Sepsis	23 (2.0)	2285 (1.2)	1.705 [1.126–2.582]	0.012

*BIPM* biapenem, *CI* confidence interval, *CIP* ciprofloxacin, *C/T* ceftolozane–tazobactam, *DOR* doripenem, *FAERS* FDA Adverse Event Reporting System, *FEP* cefepime, *GRNX* garenoxacin, *LVX* levofloxacin, *MEM* meropenem, *MXF* moxifloxacin, *ROR* reporting odds ratio, *STFX* sitafloxacin, *TFLX* tosufloxacin, *TZP* tazobactam/piperacillin.

*Data are presented as median [interquartile range].

### Retrospective observational study

#### Patient characteristics

**Figure 2:**
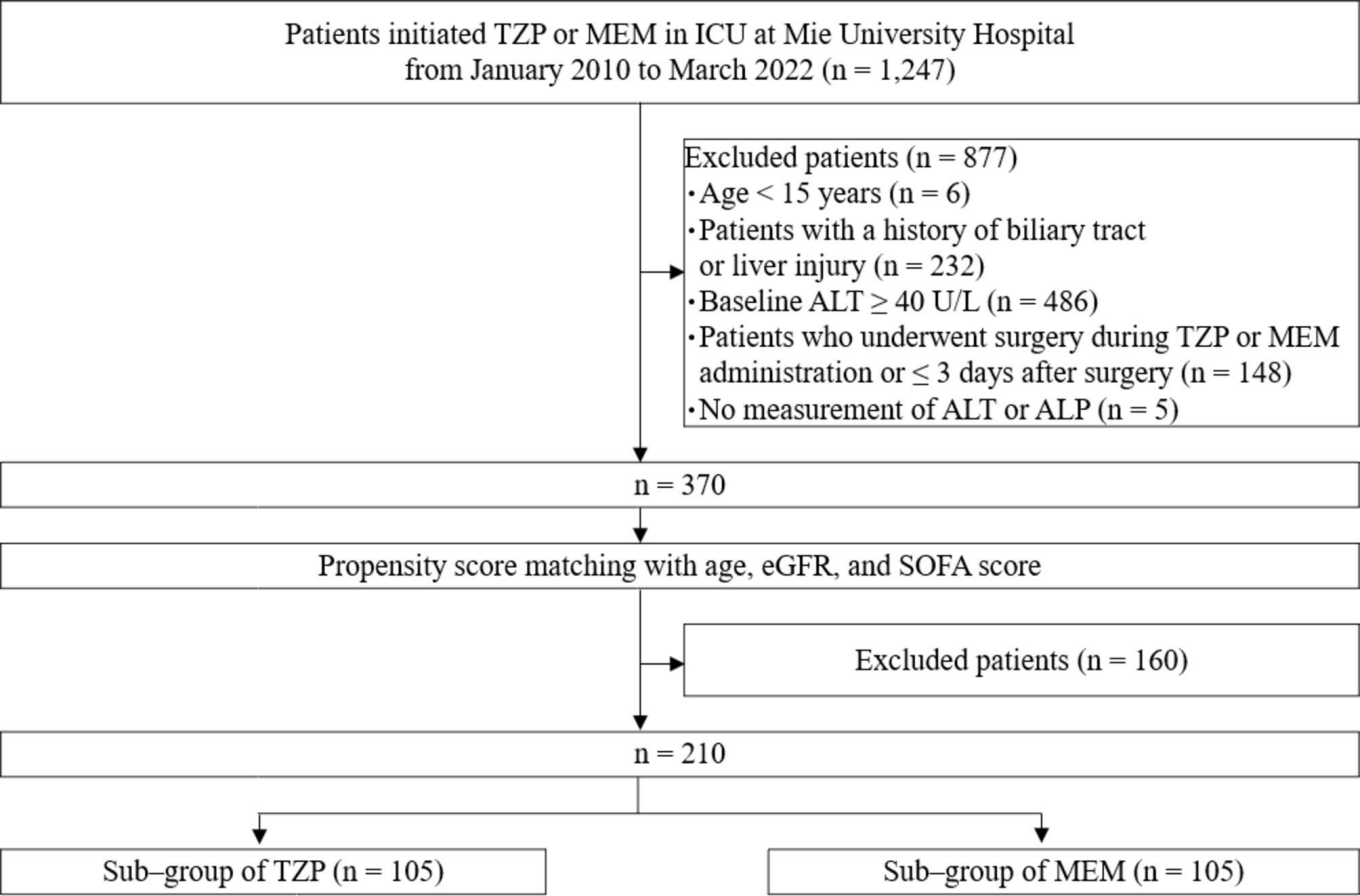
Flow chart of patient selection in the retrospective observational study. *ALT* alanine aminotransferase, *ALP* alkaline phosphatase, *eGFR* estimated glomerular filtration rate, *ICU* intensive care unit, *MEM* meropenem, *SOFA* sequential organ failure assessment, *TZP* tazobactam/piperacillin. eGFR [mL/min/1.73 m^2^] = 194 × serum creatinine^−1.094^ × age^−0.287^ (× 0.739 if female)^86^.

During the study period, 1247 patients received TZP or MEM in ICU. Figure 2 shows a flowchart of the patient selection process. The exclusion criteria were as follows: age < 15 years (n = 6), patients with a history of biliary tract or liver injury including hepatitis virus infection (n = 232), alanine aminotransferase (ALT) ≥ 40 U/L (n = 486), patients who underwent surgery during TZP or MEM administration or ≤ 3 days after surgery (n = 148), and no measurement of ALT or alkaline phosphatase (ALP) (n = 5). After conducting PSM analysis with age, estimated glomerular filtration rate (eGFR), and sequential organ failure assessment (SOFA) score, 210 patients were enrolled in this retrospective observational study. Table 2 provides a summary of the baseline characteristics of the patients and concomitant medication. None of the patients were using supplements or health foods. Liver injury occurred in of 44 (42%) patients receiving TZP and 38 (36%) patients receiving MEM. The median (interquartile range [IQR]) age, body weight, and administration period were 72.0 (66.0–79.3) years, 57.7 (48.0–67.7) kg, and 8.0 (5.0–13.3) days, respectively. No difference was observed in patient’s characteristics between TZP and MEM. Sources of infection and blood culture identification were shown in Supplementary 1 and 2.

**Table 2:** Patient characteristics in the retrospective observational study.

	All (n = 210)	TZP (n = 105)	MEM (n = 105)	*p*-value
	n (%)	n (%)	n (%)	
Age*, years	72.0 [66.0 to 79.3]	72.0 [65.0 to 80.5]	72.0 [67.0 to 78.0]	0.833
Male	145 (69.0)	74 (70.5)	71 (67.6)	0.765
Body weight*, kg	57.7 [48.0 to 67.7]	57.2 [50.0 to 68.2]	58.1 [47.2 to 67.3]	0.481
BMI*, kg/m^2^	22.3 [18.7 to 25.6]	22.9 [18.7 to 25.9]	22.0 [18.6 to 25.1]	0.333
Administration period*, day	8.0 [5.0 to 13.3]	8.0 [5.0 to 14.0]	7.0 [5.0 to 11.5]	0.690
Administration period ≥ 7 days	134 (63.8)	65 (61.9)	69 (65.7)	0.566
Alcohol drinking history	59 (28.1)	26 (24.8)	33 (31.4)	0.357
SOFA score*	7.0 [5.0 to 10.0]	7.0 [5.0 to 10.5]	7.0 [4.5 to 10.0]	0.829
PSI*	5.0 [4.0 to 5.0]	5.0 [4.0 to 5.0]	5.0 [4.0 to 5.5]	0.623
Alb*, g/dL	2.9 [2.5 to 3.4]	2.9 [2.5 to 3.3]	2.9 [2.5 to 3.4]	0.933
ALP*, U/L	247.0 [186.0 to 348.8]	202.0 [121.0 to 338.0]	242.5 [192.1 to 333.0]	0.082
ALT*, U/L	20.0 [14.0 to 28.0]	21.5 [15.0 to 30.0]	20.0 [14.0 to 28.0]	0.811
ALBI score*	− 1.6 [− 2.0 to to − 1.3]	− 1.6 [− 2.0 to − 1.3]	− 1.7 [− 2.0 to − 1.4]	0.540
FIB to 4 score*	3.0 [2.0 to 6.4]	2.7 [2.0 to 5.4]	3.3 [1.9 to 7.0]	0.172
AST*, U/L	33.0 [22.0 to 49.0]	35.0 [21.0 to 51.0]	32.0 [23.0 to 46.0]	0.613
T-Bil*, mg/dL	0.9 [0.5 to 1.5]	0.9 [0.5 to 1.5]	30.2 [19.3 to 47.1]	0.640
BUN*, mg/dL	28.3 [18.5 to 46.3]	25.1 [17.2 to 45.9]	0.8 [0.5 to 1.6]	0.348
eGFR*, mL/min/1.73 m^2^	50.1 [23.1 to 85.9]	50.3 [22.1 to 86.2]	50.0 [23.9 to 84.2]	0.515
ECMO	24 (11.4)	12 (11.4)	12 (11.4)	1.000
HD	16 (7.6)	9 (8.6)	7 (6.7)	0.398
CHDF	50 (23.8)	23 (21.9)	27 (25.7)	0.314
CRP*, mg/dL	10.7 [5.9 to 19.5]	10.0 [5.8 to 17.5]	11.4 [6.2 to 22.7]	0.224
Eosinophil*, /μL	25.0 [0.0 to 140.0]	10.0 [0.0 to 100.0]	60.0 [10.0 to 155.0]	< 0.001
Dose per day*, g per day	–	13.5 [9.0 to 13.5]	2.0 [1.5 to 3.0]	–
Dose/kg per day*, g/kg per day	–	0.2 [0.2 to 0.3]	0.04 [0.02 to 0.05]	–
Macrolide	3 (1.4)	2 (1.9)	1 (1.0)	1.000
SXT	10 (4.8)	2 (1.9)	8 (7.6)	0.101
Carbamazepine	3 (1.4)	2 (1.9)	1 (1.0)	1.000
Valproic acid	3 (1.4)	3 (2.9)	0 (0.0)	0.246
Acetaminophen	47 (22.4)	30 (28.6)	17 (16.2)	0.046
Acetaminophen dose	500.0 [400.0 to 1000.0]	800.0 [400.0 to 1000.0]	400.0 [400.0 to 1100.0]	0.225
Amiodarone	10 (4.8)	6 (5.7)	4 (3.8)	0.748
Statin	8 (3.8)	8 (7.6)	0 (0.0)	0.007
Propofol	23 (11.0)	13 (12.4)	10 (9.5)	0.659
RIF	0 (0.0)	0 (0.0)	0 (0.0)	1.000
NSAIDs	20 (9.5)	9 (8.6)	11 (10.5)	0.815
Opioids	107 (51.0)	55 (52.4)	52 (49.5)	0.783
VAN	53 (25.2)	21 (20.0)	32 (30.5)	0.084
TEI	7 (3.3)	4 (3.8)	3 (2.9)	1.000
LIN	18 (8.6)	7 (6.7)	11 (10.5)	0.230
DAP	11 (5.2)	4 (3.8)	7 (6.7)	0.269
CAS	1 (0.5)	1 (1.0)	0 (0.0)	1.000
MFG	1 (0.5)	1 (1.0)	0 (0.0)	1.000

ALBI score = (log_10_ T-Bil × 0.66) + (− 0.085 × Alb)^84^.

FIB-4 score = age × AST/platelet × (ALT)^1/2^^85^.

eGFR [mL/min/1.73 m^2^] = 194 × serum creatinine^−1.094^ × age^−0.287^ (× 0.739 if female)^86^.

Chi–squared test or Mann–Whitney U test.

*Alb* serum albumin, *ALBI* albumin–bilirubin, *ALP* alkaline phosphatase, *ALT* alanine aminotransferase, *AST* aspartate aminotransferase, *BMI* body mass index, *BUN* blood urea nitrogen, *CHDF* continuous hemodiafiltration, *CAS* caspofungin, *CRP* C-reactive protein, *DAP* daptomycin, *ECMO* extracorporeal membrane oxygenation, *eGFR* estimated glomerular filtration rate, *FIB-4* Fibrosis-4, *HD* hemodialysis, *LIN* linezolid, *MFG* micafungin, *MEM* meropenem, *NSAIDs* non-steroidal anti-inflammatory drugs, *PSI* pneumonia severity index, *TEI* teicoplanin, *RIF* rifampicin, *SOFA* sequential organ failure assessment, *SXT* sulfamethoxazole/trimethoprim, *T-Bil* total bilirubin, *VAN* vancomycin.

*Data are presented as median [interquartile range].

#### The biochemical classification

**Figure 3:**
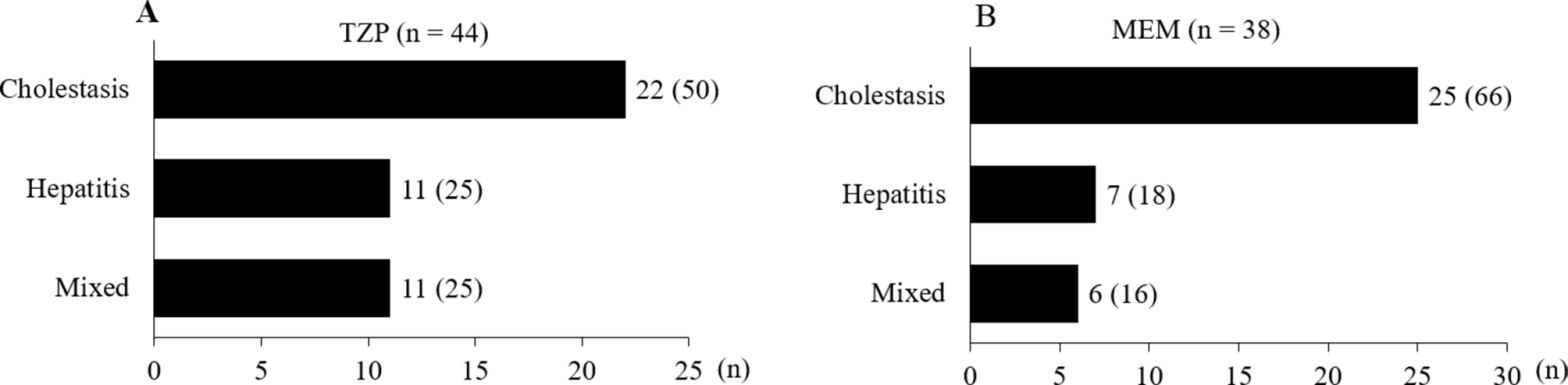
Classification of liver injury using the RUCAM of TZP (**A**). Classification of liver injury using the RUCAM of MEM (**B**). Among 82 patients developed liver injury, 47 (57.3%) developed classified as cholestasis. For TZP, 22 patients (50%) had cholestasis, 11 patients (25%) had hepatitis, and 11 patients (25%) had mixed cholestasis/hepatitis; for MEM, 25 patients (66%) had cholestasis, 7 patients (18%) had hepatitis, and 6 patients (16%) had mixed cholestasis/hepatitis (this figure). According to the RUCAM, the liver injury in this study was possible or probable induced by TZP or MEM: TZP 5.0 (4.0–6.0) and MEM 6.0 (5.0–7.0) (data not shown). *MEM* meropenem, *RUCAM* Roussel Uclaf Causality Assessment Method, *TZP* tazobactam/piperacillin.

Figure 3 shows the biochemical classification of liver injury based on the Roussel Uclaf Causality Assessment Method (RUCAM) (TZP; n = 44, MEM; n = 38). Among 82 patients developed liver injury, 47 (57.3%) developed classified as cholestasis. For TZP, 22 patients (50%) had cholestasis, 11 patients (25%) had hepatitis, and 11 patients (25%) had mixed cholestasis/hepatitis; for MEM, 25 patients (66%) had cholestasis, 7 patients (18%) had hepatitis, and 6 patients (16%) had mixed cholestasis/hepatitis (Fig. 3). According to the RUCAM, the liver injury in this study was possible or probable induced by TZP or MEM: TZP 5.0 (4.0–6.0) and MEM 6.0 (5.0–7.0) (data not shown).

#### Univariate and multivariate logistic regression analysis for TZP or MEM

The cut-off values and area under the receiver operating characteristic curve (AUC) of administration period was 7 days (AUC, 0.70; *p* = 0.001). The univariate logistic regression analysis determined that male, body weight, administration period ≥ 7 days, SOFA score, pneumonia severity index (PSI), ALT, aspartate aminotransferase (AST), and total bilirubin (T-Bil) were potential independent variables in liver injury. The final model using the stepwise forward selection method included male (odds ratio [OR] 2.026, 95% CI 1.112–3.621, *p* = 0.046), administration period ≥ 7 days (OR 4.558, 95% CI 2.360–9.204,* p* < 0.001), and ALT (OR 1.033, 95% CI 1.003–1.066,* p* = 0.031) (adjusted coefficient of determination = 0.124, *p* < 0.001; Table 3).

**Table 3:** Univariate and multivariate logistic regression analysis of TZP or MEM in the retrospective observational study.

	TZP or MEM (n = 210)			
	Univariate analysis		Multivariate analysis (variable selection)	
	OR [95% CI]	*p*-value	OR [95% CI]	*p*-value
Age, years	0.987 [0.967–1.007]	0.197	–	–
Male	2.140 [1.137–4.028]	0.016	2.026 [1.112–3.621]	0.046
Body weight, kg	1.021 [1.003–1.039]	0.014	–	–
BMI, kg/m^2^	1.043 [0.992–1.097]	0.084	–	–
Administration period ≥ 7 days	5.229 [2.748–10.441]	< 0.001	4.558 [2.360–9.204]	< 0.001
Alcohol drinking history	0.966 [0.511–1.824]	0.914	–	–
SOFA score	1.119 [1.042–1.201]	0.001	–	–
PSI	0.708 [0.532–0.942]	0.015	–	–
Alb, g/dL	0.945 [0.550–1.623]	0.836	–	–
ALP, U/L	1.000 [0.998–1.000]	0.667	–	–
ALT, U/L	1.048 [1.017–1.080]	0.002	1.033 [1.003–1.066]	0.031
ALBI score	1.369 [0.762–2.456]	0.291	–	–
FIB-4 score	1.018 [0.980–1.058]	0.344	–	–
AST, U/L	1.011 [1.000–1.023]	0.049	–	–
T-Bil, mg/dL	1.111 [0.992–1.244]	0.043	–	–
BUN, mg/dL	1.002 [0.991–1.013]	0.759	–	–
eGFR, mL/min/1.73m^2^	1.003 [0.997–1.010]	0.280	–	–
CRP, mg/dL	0.993 [0.962–1.025]	0.651	–	–
Eosinophil, /μL	0.998 [0.996–1.000]	0.074	–	–
Co–administered drugs	0.887 [0.469–1.678]	0.712	–	–
Acetaminophen dose, mg per day	1.121 [0.989–1.201]	0.189	–	–
MEM	0.758 [0.436–1.317]	0.324	–	–

Adjusted coefficient of determination = 0.124 (*p* < 0.001).

ALBI score = (log_10_ T-Bil × 0.66) + (− 0.085 × Alb)^84^.

FIB-4 score = age × AST/platelet × (ALT)^1/2^^85^.

eGFR [mL/min/1.73 m^2^] = 194 × serum creatinine^−1.094^ × age^−0.287^ (× 0.739 if female)^86^.

Administration period ≥ 7 days was coded 1, and < 7 days was coded 0, and univariate and multivariate binary logistic regression analysis was conducted.

Variables with *p* < 0.05 were included in the multivariate model. Using the stepwise forward selection method, potential independent variables were further examined to construct the final model.

In the multivariate model, all independent variables were considered as candidates. The final model was constructed using the stepwise forward selection method, with a selection criterion of *p* < 0.05. Selected variables are presented as OR [95% CI] and *p*-value, while unselected variables are presented as “–”.

*Alb* serum albumin, *ALBI* albumin–bilirubin, *ALP* alkaline phosphatase, *ALT* alanine aminotransferase, *AST* aspartate aminotransferase, *BMI* body mass index, *BUN* blood urea nitrogen, *CI* confidence interval, *CRP* C-reactive protein, *eGFR* estimated glomerular filtration rate, *FIB-4* Fibrosis-4, *MEM* meropenem, *OR* odds ratio, *PSI* pneumonia severity index, *SOFA* sequential organ failure assessment, *T-Bil* total bilirubin, *TZP* tazobactam/piperacillin.

#### Sub-group analysis for TZP

The cut-off value and AUC of administration period was 7 days (AUC, 0.69; *p* = 0.009). The univariate logistic regression analysis demonstrated that administration period ≥ 7 days, SOFA score, ALT, AST, and eosinophil were selected as potential independent variables in liver injury. By using the stepwise forward selection method, the final model included administration period ≥ 7 days (OR 3.862, 95% CI 1.566–10.182, *p* = 0.003) and ALT (OR 1.064, 95% CI 1.019–1.116,* p* = 0.004) (adjusted coefficient of determination = 0.144, *p* = 0.044; Table 4). When the interaction term was introduced, no significant interactions were found between the independent variables.

**Table 4:** Univariate and multivariate logistic regression analysis divided TZP and MEM in the retrospective observational study.

	TZP (n = 105)				MEM (n = 105)			
	Univariate analysis		Multivariate analysis (variable selection)		Univariate analysis		Multivariate analysis (variable selection)	
	OR [95% CI]	*p*-value	OR [95% CI]	*p*-value	OR [95% CI]	*p*-value	OR [95% CI]	*p*-value
Age, years	0.985 [0.959–1.012]	0.278	–	–	0.990 [0.961–1.020]	0.515	–	–
Male	1.398 [0.586–3.336]	0.446	–	–	2.989 [1.015–7.764]	0.018	3.582 [1.334–10.575]	0.011
Body weight, kg	1.017 [0.994–1.399]	0.120	–	–	1.023 [0.994–1.053]	0.121	–	–
BMI, kg/m^2^	1.039 [0.975–1.107]	0.210	–	–	1.028 [0.942–1.120]	0.015	–	–
Administration period ≥ 7 days	4.465 [1.879–11.429]	0.001	3.862 [1.566–10.182]	0.003	6.379 [2.473–18.862]	< 0.001	6.446 [2.430–19.562]	< 0.001
Alcohol drinking history	0.593 [0.227–1.545]	0.277	–	–	1.164 [0.472–2.872]	0.742	–	–
SOFA score	1.164 [1.044–1.298]	0.004	–	–	1.082 [0.983–1.190]	0.103	–	–
PSI	0.797 [0.532–1.195]	0.269	–	–	0.608 [0.441–0.922]	0.014	–	–
Alb, g/dL	0.669 [0.313–1.433]	0.296	–	–	1.415 [0.643–3.116]	0.387	–	–
ALP, U/L	1.000 [0.998–1.001]	0.871	–	–	1.001 [0.996–1.005]	0.746	–	–
ALT, U/L	1.063 [1.019–1.108]	0.002	1.064 [1.019–1.116]	0.004	1.035 [0.988–1.084]	0.146	–	–
ALBI score	1.937 [0.844–4.446]	0.109	–	–	0.848 [0.360–1.996]	0.705	–	–
FIB-4 score	1.029 [0.953–1.111]	0.464	–	–	1.003 [0.965–1.042]	0.880	–	–
AST, U/L	1.020 [1.003–1.037]	0.013	–	–	1.001 [0.984–1.018]	0.922	–	–
T-Bil, mg/dL	1.096 [0.960–1.251]	0.128	–	–	1.127 [0.919–1.381]	0.240	–	–
BUN, mg/dL	1.010 [0.996–1.025]	0.160	–	–	0.987 [0.969–1.005]	0.137	–	–
eGFR, mL/min/1.73m^2^	0.998 [0.989–1.007]	0.718	–	–	1.009 [1.000–1.018]	0.049	–	–
CRP, mg/dL	0.995 [0.946–1.045]	0.831	–	–	0.992 [0.951–1.035]	0.012	–	–
Eosinophil, /μL	0.995 [0.991–1.000]	0.020	–	–	1.000 [0.996–1.030]	0.862	–	–
Dose per day, g per day	1.036 [0.928–1.156]	0.532	–	–	1.093 [0.701–1.705]	0.694	–	–
Dose/kg per day, g/kg per day	0.116 [0.001–10.617]	0.343	–	–	0.925 [0.750–1.141]	0.457	–	–
Co–administered drugs	1.143 [0.483–2.702]	0.762	–	–	1.000 [0.841–1.021]	0.652	–	–
Acetaminophen dose, mg per day	1.000 [0.998–1.002]	0.916	–	–	0.999 [0.997–1.002]	0.518	–	–

TZP; adjusted coefficient of determination = 0.144 (*p* = 0.044).

MEM; adjusted coefficient of determination = 0.162 (*p* < 0.001).

ALBI score = (log_10_ T-Bil × 0.66) + (− 0.085 × Alb)^84^.

FIB-4 score = age × AST/platelet × (ALT)^1/2^^85^.

eGFR [mL/min/1.73 m^2^] = 194 × serum creatinine^−1.094^ × age^−0.287^ (× 0.739 if female)^86^.

Administration period ≥ 7 days was coded 1, and < 7 days was coded 0, and univariate and multivariate binary logistic regression analysis was conducted.

Variables with *p* < 0.05 were included in the multivariate model. Using the stepwise forward selection method, potential independent variables were further examined to construct the final model.

In the multivariate model, all independent variables were considered as candidates. The final model was constructed using the stepwise forward selection method, with a selection criterion of *p* < 0.05. Selected variables are presented as OR [95% CI] and *p*-value, while unselected variables are presented as “–”.

*Alb* serum albumin, *ALBI* albumin–bilirubin, *ALP* alkaline phosphatase, *ALT* alanine aminotransferase, *AST* aspartate aminotransferase, *BMI* body mass index, *BUN* blood urea nitrogen, *CI* confidence interval, *CRP* C-reactive protein, *eGFR* estimated glomerular filtration rate, *FIB-4* Fibrosis-4, *MEM* meropenem, *OR* odds ratio, *PSI* pneumonia severity index, *SOFA* sequential organ failure assessment, *T-Bil* total bilirubin, *TZP* tazobactam/piperacillin.

#### Sub-group analysis for MEM

The cut–off value and AUC of administration period was 7 days (AUC, 0.72;* p* = 0.035). The univariate logistic regression analysis demonstrated that male, body mass index (BMI), administration period ≥ 7 days, PSI, eGFR, and C-reactive protein (CRP) were selected as potential independent variables in liver injury. By using the stepwise forward selection method, the final model included male (OR 3.582, 95% CI 1.334–10.575, *p* = 0.011) and administration period ≥ 7 days (OR 6.446, 95% CI 2.430–19.562, *p* < 0.001) (adjusted coefficient of determination = 0.162, *p* < 0.001; Table 4). When the interaction term was introduced, no significant interactions were found between the independent variables.

#### Administration period

For TZP, the administration period increased as the Common Terminology Criteria for Adverse Events (CTCAE) grade of liver injury increased (grade 0, 6.0 [5.0–12.0] days; grade 1, 10.0 [6.0–14.0] days; grade 2, 12.5 [7.5–15.3] days; grade ≥ 3, 18.0 [18.0–20.0] days;* p* = 0.002). For MEM, the administration period was significantly longer in patients with severe liver injury, similar to TZP (grade 0, 6.0 [5.0–9.0] days; grade 1, 10.0 [7.0–12.5] days; grade 2, 11.5 [7.0–14.5] days; grade ≥ 3, 11.0 [7.0–15.8] days; *p* = 0.002) (data not shown).

### Comparison of patient background by sex

Supplementary 3 shows the comparison of patient background by sex in patients receiving TZP or MEM. Body weight, alcohol drinking history, and opioids administration were significantly higher in male than in female (male vs. female: body weight; 59.7 kg vs. 51.1 kg, *p* < 0.001; alcohol drinking history, 44% vs. 9%, *p* < 0.001; opioids, 58% vs. 35%, *p* = 0.003).

### Sub-group analysis of non-septic patients

We conducted a sub-group analysis of non-septic patients (TZP; n = 105, MEM; n = 206). Supplementary 4 provides a summary of the baseline characteristics of the patients and concomitant medication. Liver injury occurred in 43 (41%) patients receiving TZP and 72 (34%) patients receiving MEM.

The univariate logistic regression analysis of TZP or MEM determined that male, body weight, administration period ≥ 7 days, SOFA score, and PSI were potential independent variables in liver injury. The final model using the stepwise forward selection method included administration period ≥ 7 days (OR 3.977, 95% CI 2.394–6.777,* p* < 0.001) (adjusted coefficient of determination = 0.104, *p* < 0.001; Supplementary 5).

The univariate logistic regression analysis of TZP demonstrated that administration period ≥ 7 days, SOFA score, ALT, AST, eosinophil, and co-administered drugs were selected as potential independent variables in liver injury. By using the stepwise forward selection method, the final model included administration period ≥ 7 days (OR 4.170, 95% CI 1.676–11.265, *p* = 0.002) and ALT (OR 1.064, 95% CI 1.019–1.115,* p* = 0.004) (adjusted coefficient of determination = 0.148, *p* < 0.001; Supplementary 6).

The univariate logistic regression analysis of MEM demonstrated that male, body weight, administration period ≥ 7 days, PSI, ALP, AST, eGFR, and dose per day were selected as potential independent variables in liver injury. By using the stepwise forward selection method, the final model included male (OR 2.365, 95% CI 1.208–4.797, *p* = 0.014), administration period ≥ 7 days (OR 4.360, 95% CI 2.246–8.887, *p* < 0.001), and eGFR (OR 1.012, 95% CI 1.006–1.020, *p* < 0.001) (adjusted coefficient of determination = 0.129, *p* < 0.001; Supplementary 6).

## Discussion

The retrospective observational study revealed that the incidence of liver injury increased in association with the administration period of TZP or MEM. An expanded administration period ≥ 7 days increased the incidence of liver injury. The administration period was prolonged in patients receiving TZP or MEM and corresponded with CTCAE grade of liver injury. These findings suggest that early antibiotic change reduces the development of liver injury. In the FAERS database study, sepsis was one of the risk factors on liver injury. These findings indicated that the baseline condition contributed to the liver injury. Furthermore, MEM-induced liver injury was significantly higher in males; this finding is consistent with the FAERS database study.

Antibiotics are one of the common causes of drug-induced liver injury^15,16^. The liver receives most of its blood supply (approximately 70%) from the intestine via portal circulation and is constantly exposed to gut-derived factors including bacterial products and inflammatory cytokines. Therefore, reducing bacterial density, eliminating target harmful bacteria, inhibiting secondary bacterial proliferation, and reducing bacterial translocation by the liver are considered the pharmacological mechanisms of drug-induced liver injury.

Antibiotic therapy aims to eliminate pathogenic bacteria; however, this microbial clearance also reduces beneficial commensal bacteria, and this situation has important pathological implications for the liver^17,18^. The risk of drug-induced liver injury caused by microbiota dysbiosis has been recognized a multifactorial process. Antibiotics directly affect the structure and diversity of gut microbiota, which alters metabolites. The depletion of probiotics after antibiotics interference can reduce the efficacy of hepatoprotective agents and leads to the development of liver injury^19^. *Lactobacillus* and *Bifidobacterium* spp., which play significant roles in regulating intestinal flora balance, reduce oxidative stress and inflammation and improve liver pathological changes^20^. By contrast, it has been reported that cefoxitin significantly reduces gut microbiota diversity and increases the levels of pathogenic bacteria such as *Firmicutes*, *Tendericutes*, and *Vibrio*, which cause damage to the intestinal barrier^21^. In other words, the sufficient decreases in beneficial intestinal bacteria such as *Lactobacillus* spp. and *Bifidobacterium* spp. and the sufficient increase in pathogenic bacteria, including *Enterococcus* spp., can aggravate the progression of liver injury^20,22^. Therefore, a follow-up liver function test is essential during the administration of antibiotics that affect intestinal microorganisms and their metabolic activities.

TZP- or MEM-associated cholestasis was seen in many patients in this retrospective observational study. Cholestatic injury accounts for approximately 30% of drug-induced liver injuries^23^. Most cases are mild and manageable and occasionally leads to vanishing bile duct syndrome^24^. Cholestatic hepatitis caused by carbapenems is probably immunoallergic and resembles the rare and clinically apparent liver injury that has been linked to penicillin and cephalosporins. In a Spanish cohort, patients older than 55 years were significantly more likely to develop a cholestatic or mixed liver injury. Given that the median age of this study population is 72.0 years, older age may have contributed to the development of liver injury^25^.

Short-course therapy (Community-acquired pneumonia, 3 or 5 days^26–34^; hospital-acquired/ventilator-associated pneumonia, 7–8 day^35,36^; complicated urinary tract infections/pyelonephritis, 5 or 7 days^37–42^; complicated/postoperative intraabdominal infections; 4 or 8 days^43,44^; Gram-negative bacteremia, 7 days^45^; acute exacerbation of chronic bronchitis/chronic obstructive pulmonary disease, ≤ 5 days^46^; acute bacterial skin and skin structure infections (cellulitis/major abscess), 5–6 days^47–49^; chronic osteomyelitis, 42 days^50^; and empiric neutropenic fever, afebrile and stable × 72 h)^51^ is just as effective as longer courses with point of clinical success, fewer adverse events, and/or diminished emergence of resistance at the site of infection^52^. In clinical settings, a 3-day incubation period is sufficient to reveal the result of a blood culture test^53^. Therefore, antimicrobial de-escalation is a treatment strategy in early adequate antimicrobial therapy. A previous study revealed that de-escalation is not associated with adverse consequences compared with treatment with broad-spectrum antibiotics (mortality or clinical failure)^54^. In addition, previous studies have revealed that antimicrobial de-escalation seems to be significantly associated with better clinical outcomes compared with non-antimicrobial de-escalation^55,56^. Hence, clinicians should consider switching from TZP or MEM to other antibiotics within 7 days. By contrast, the careful monitoring of liver function is needed for patients who require the prolonged administration of TZP or MEM. A reduction in the length of antibiotic administration is a potentially viable strategy to minimize the consequences of antibiotic overuse in critical care, including antibiotic resistance, adverse event, clostridium difficile colitis, and costs^57^. Bacterial resistance decreases the chance of early adequate therapy^58^ and are associated with mortality^59,60^.

The elevation of ALT from baseline may be positively associated with drug-induced liver injury. This finding was consistent with previous studies^61,62^. A positive correlation between ALT levels and hepatocyte necrosis also existed in an animal study^63^. Oxidative stress, which causes necrosis, is removed by Kupffer cells in the liver^64^, thus suggesting that Kupffer cell function and the number of Kupffer cells may be involved in the development of drug-induced liver injury^61^. However, it is unclear whether TZP- or MEM-induced liver injuries occur in similar phenomena. Therefore, the mechanism of TZP- or MEM-induced liver injury needs further research.

Heavy drinkers are likely to develop alcoholic hepatitis, which is associated with impaired liver function due to a severe inflammatory response^65–68^. It was reported that men tend to consume more alcohol and experience alcohol-induced physical impairment compared with women^69^. Notably, alcohol history has been found to be substantially present in men in our study population (Supplementary 3), thus suggesting that men are at a high risk of liver injury. It has been reported that amoxicillin–induced liver injury is also more frequent in men than in women^70^.

Sepsis is defined as a life-threatening organ dysfunction caused by the dysregulated response of the host to infection. Acetaminophen-induced liver injury reported that a SOFA score > 7 points predict organ failure^71^. The liver plays a key role in the development of the inflammatory response after bacterial infection. Kupffer cells remove bacteria from the circulation, ingest endotoxin, and modulate the immune response through the release of proinflammatory mediators^3^. These factors may contribute to the development of hepatic dysfunction during sepsis.

There are several limitations of this study. First, we extracted reports of liver injury that occurred during the administration of broad-spectrum antibiotics by using the term “drug-induced liver injury”, which was derived from Medical Dictionary for Regulatory Activities (MedDRA) terminology; however, the causal relationship between events, medications, and severity of liver injury remains uncertain. Since there is no certainty that a reported event was actually caused by the drug, risk signals indicate increased risk for reporting but not the true risk. Second, information on the concomitant use of drugs that potentially interact with TZP or MEM was lacking in the FAERS database. Third, given that the FAERS database did not include clinical laboratory data such as serum creatinine level and alanine aminotransferase, we could not evaluate renal and liver functions as risk factors of antibiotics-induced liver injury. Fourth, in FAERS database, every adverse event observed in clinical setting is not always reported to regulatory authorities. Reporting bias and lack of a denominator and confounder adjustment were observed. This was a single-center retrospective observational study; thus, it was difficult to avoid the influence of unknown confounding factors. Fifth, among the carbapenem group, only two patients received doripenem and one patient received biapenem. Most patients were administered MEM; therefore, we extracted only patients receiving MEM. In the future, further study is needed to clarify the differences in carbapenem-treated patients. Sixth, since critically ill patients were received numerous medications and often experienced the change of drug dosing and medication regimens, it is difficult to exclude the patients who used additional drugs other than TZP or MEM. Seventh, although routine laboratory biochemistry helps to detect liver injury but remains of limited value in evaluating hepatic function. Eighth, we did not analyze the difference of clinical efficacy and adverse events between patients with or without de-escalation. Finally, prolonged infusions attain the pharmacodynamic efficacy target defined for *β*-lactam antibiotics more effectively than short infusions without increasing adverse events^72^, however, in our observational study, none of the patients were received antibiotics by such way. Therefore, to clarify the association of these, further study is needed. The development of novel techniques to assess hepatic function at the bedside potentially may help to standardize the definition of acute liver injury^73^.

## Conclusions

Our study was the first to demonstrate that a prolonged administration period ≥ 7 days could increase the risk of liver injury in patients receiving TZP or MEM. Our findings recommend a periodical monitoring of liver function in ICU patients receiving TZP or MEM, particularly in those with multiple risk factors for the development of liver injury. The findings of this study provide useful information for the appropriate use of TZP or MEM. Further studies are required to fully understand the mechanism of TZP- or MEM-induced liver injury.

## Materials and methods

### FAERS database study

The FAERS database generated from post-marketing spontaneous pharmacovigilance databases was used for this study. The FAERS database includes ˃ 1.4 million cases of adverse events reported worldwide after January 2004 and consists of seven data tables: patient demographic and administrative information, drug and biologic information, adverse events, patient outcomes, report sources, start end dates of drug therapy, and indications for use/diagnosis^74^. The source of the databases complies with the guidelines set by the International Council for Harmonisation of Technical Requirements for Pharmaceuticals for Human Use (ICH) and the databases adhere to the ICH-standardized adverse event information guidelines^75^. For the current study, adverse event reports were downloaded from the website of the US Food and Drug Association (FDA) (https://www.fda.gov/; accessed on January 22, 2023). Reports with the same case number were identified as duplicate reports, and the most recent reports were used, as recommended by the FDA^76^. Data were extracted from the FAERS database between January 2013 and September 2022. We collected reports of the following antibiotics, which have broad-spectrum use in empiric therapy in the ICU^77^: the fourth-generation cephalosporin (cefepime), carbapenem (doripenem, biapenem, and MEM), *β*-lactam and *β*-lactamase inhibitor combination (tazobactam/ceftolozane and TZP), and quinolone (garenoxacin, sitafloxacin, ciprofloxacin, tosufloxacin, moxifloxacin, and levofloxacin). The collected data included the case number, drug name, adverse event name, start date of administration, end date of administration, date of development of the adverse event, sex, age, and medical history of sepsis (10040054), which were derived from MedDRA terminology. The sample code was provided in Supplementary 7.

### Adverse events detection

Adverse events were coded according to preferred terms (drug-induced liver injury; 10072268) derived from MedDRA terminology. Event reports were identified using the standardized MedDRA query (version 26.0). Reports with development of liver injury during antibiotics administration were classified as “liver injury group” and other reports as “non-liver injury group”.

### Retrospective observational study

Data from patients receiving TZP or MEM in ICU of Mie University Hospital from January 2010 to March 2022 were analyzed. The exclusion criteria were age < 15 years, patients with a history of biliary tract or liver injury, baseline ALT ≥ 40 U/L, patients who underwent surgery during TZP or MEM administration or ≤ 3 days after surgery, and no measurement of ALT or ALP. A retrospective observational study was performed to obtain the following demographic data: sex; age; height; body weight; BMI; daily dose and treatment duration of MEM and TZP; baseline clinical laboratory data (serum albumin [Alb], ALP, ALT, albumin–bilirubin [ALBI] grade, Fibrosis-4 [FIB-4] score, AST, T-Bil, blood urea nitrogen, serum creatinine [SCr], eGFR, CRP, and eosinophil); and medical records of extracorporeal membrane oxygenation, hemodialysis, and continuous hemodiafiltration. The SOFA score and PSI were extracted for the evaluation of infection severity^78,79^. As the mortality rate is directly related to the degree of organ dysfunction, it is evident that it must also be related to the SOFA score for each organ system^80^. PSI scoring system is one of the most widely used scoring tools to evaluate the condition and prognosis of community acquired pneumonia patients^81^. We collected information on concomitant systematically administered medications that could cause drug-induced liver injury (macrolide, sulfamethoxazole/trimethoprim, carbamazepine, valproic acid, acetaminophen, amiodarone, statin, propofol, rifampicin, non-steroidal anti-inflammatory drugs, and opioids)^82,83^, other antibiotics, antifungal drugs, supplements, and health foods. Acetaminophen dose was also extracted. The ALBI score and FIB-4 score were calculated using the following formula: ALBI score = (log_10_ T-Bil × 0.66) + (− 0.085 × Alb)^84^, FIB-4 score = age × AST/platelet × (ALT)^1/2^^85^. The eGFR was calculated using the prediction equation for Japanese patients: eGFR (mL/min/1.73 m^2^) = 194 × SCr^−1.094^ × age^−0.287^ (× 0.739 if female)^86^. A subgroup analysis of non-septic cases was also conducted.

### PSM analysis

Age, eGFR, and SOFA score were considered potential confounders in patients receiving TZP or MEM, and these were adjusted using PSM analysis. Propensity scores were calculated using bivariate logistic regression by using a 1:1 case–control match with a caliper value of 0.1 (one-to-one nearest-neighbor matching). The standardized difference (10% or 0.1) was used to compare the distribution of all paired covariates between subgroup (TZP or MEM).

### Liver injury evaluation

Liver injury was defined as an elevation of ALT grade on the basis of the CTCAE version 4.0 (Supplementary 8) after the initiation of TZP or MEM therapy^11^. The possibility of TZP- or MEM-induced liver injury was evaluated using the RUCAM (≤ 0 indicate that the drug is “excluded” as a cause; 1 to 2 that it is “unlikely”; 3 to 5 “possible”; 6 to 8 “probable”; and > 8, “highly probable”)^11^. The total score consists of points for 8 separate factors in 7 categories that help define the “signature” of the drug induced liver injury. These factors are: (1) time to onset (+ 1 or + 2); (2) course (− 2, 0, + 1, + 2, or + 3); (3) risk factors (0 or + 1); (4) concomitant drugs (0, − 1, − 2, or − 3); (5) nondrug causes of liver injury (− 3, − 2, 0, + 1, or + 2); (6) previous information on the hepatotoxicity of the drug (0, + 1, or + 2); and (7) response to rechallenge (− 2, 0, + 1, or + 3). The individual points range from − 3 to + 3 and the total possible score ranges from − 9 to + 14. Liver injury was classified biochemically as being hepatitis, cholestatic, mixed cholestasis/hepatitis, and other by using R value, which was defined as ALT/upper limit of normal (ULN) divided by ALP/ULN: hepatitis, ALT > 2 ULN and ALP ≤ ULN or R ≥ 5; cholestatic, ALT ≤ ULN and ALP > 2 ULN or R ≤ 2; mixed, ALT > 2 ULN, ALP > ULN, and 2 < R < 5^14^.

### Statistical analyses

Statistical analyses were performed using R software version 4.1.3 (R Core Team, 2022)^87^ and JMP Pro 16 statistical package (SAS Institute, Cary, NC, USA). Categorical data were summarized as numbers (%) and analyzed using the chi-squared test. Continuous data were summarized as medians (IQR) and analyzed using the Mann–Whitney U test. Statistical significance was defined as a two-tailed *p*-value < 0.05. Cut-off values of administration period for the development of liver injury were determined by receiver operating characteristics curve method.

#### FAERS database study

The association between clinical profiles and antibiotics-induced liver injury was evaluated using ROR with a 95% CI. A statistically significant ROR was formally defined as a lower limit of the 95% CI exceeding 1.0.

#### Retrospective observational study

Univariate and multivariate logistic regression analyses were performed to identify the independent variables for the TZP- or MEM-induced liver injury. Variables with *p*-value < 0.05 were included in the multivariate model. By using the stepwise forward selection method, potential independent variables were further examined to construct the final model. A multivariate logistic regression analysis was used to determine the multivariate regression equation for TZP- or MEM-induced liver injury. We verified these interactions by focusing on each significant independent variable. Clinical characteristics were compared and stratified by independent variables as needed. When multicollinearity was present, we selected an independent variable on the basis of its clinical relevance by sensitivity analyses. The cut-off values of continuous variables for the development of TZP- or MEM-induced liver injury was determined by using the receiver operating characteristic curve method. Missing values were handled without correction.

### Ethics approval

This study was conducted in accordance with the Declaration of Helsinki and its amendments after obtaining approval from the Clinical Research Ethics Review Committee of Mie University Hospital (No. H2022-107). For the retrospective observational study, individual informed consent was waived by the ethics committee, and an opt-out approach was used, allowing patients to decline participation. The FAERS data analysis was exempt from ethical review. The FAERS data used in the current study were de-identified (anonymous) and publicly available. The study adhered to all relevant ethical guidelines for research involving human data.

### Patents

This section is not mandatory but may be added if there are patents resulting from the work reported in this manuscript.

### Supplementary Information

Supplementary Information.

## References

[1] Tamma, P. D., Avdic, E., Li, D. X., Dzintars, K. & Cosgrove, S. E. Association of adverse events with antibiotic use in hospitalized patients. *JAMA Intern. Med.* **177**, 1308–1315 (2007). 10.1001/jamainternmed.2017.1938 PMC5710569 28604925

[2] Emmerson, M. Antibiotic usage and prescribing policies in the intensive care unit. *Intens. Care Med.* **26**, 26–30 (2000). 10.1007/s001340051115 10786955

[3] Lescot, T., Karvellas, C., Beaussier, M. & Magder, S. Acquired liver injury in the intensive care unit. *Anesthesiology* **117**, 898–904 (2012). 22854981 10.1097/ALN.0b013e318266c6df

[4] Park, J. H. *et al.* Prevalence and clinical characteristics of antibiotics associated drug induced liver injury. *Ann. Transl. Med.* **9**, 642 (2021). 33987340 10.21037/atm-20-5144 PMC8106034

[5] Gin, A. *et al.* Piperacillin-tazobactam: A beta-lactam/beta-lactamase inhibitor combination. *Expert Rev. Anti Infect. Ther.* **5**, 365–383 (2007). 17547502 10.1586/14787210.5.3.365

[6] Thomas, C., Priano, J. & Smith, T. L. Meropenem as an antidote for intentional valproic acid overdose. *Am. J. Emerg. Med.* **38**, e1–e2 (2020). 10.1016/j.ajem.2019.09.011 31980292

[7] The Infection Control Committee, Japanese Society of Intensive Care Medicine. Investigation of antimicrobial usage in ICU. *J. Jpn. Soc. Intens. Care Med.* **28**, 60–67 (2021).

[8] Akimoto, H. *et al.* Signal detection of potential hepatotoxic drugs: Case-control study using both a spontaneous reporting system and electronic medical records. *Biol. Pharm. Bull.* **44**, 1514–1523 (2021). 34602560 10.1248/bpb.b21-00407

[9] Kang, Y. *et al.* Evaluation of drug-induced liver injury developed during hospitalization using electronic health record (EHR)-based algorithm. *Allergy Asthma Immunol. Res.* **12**, 430–442 (2000). 10.4168/aair.2020.12.3.430 PMC7061161 32141257

[10] Pedraza, L. *et al.* Drug induced liver injury in geriatric patients detected by a two-hospital prospective pharmacovigilance program: A comprehensive analysis using the Roussel uclaf causality assessment method. *Front. Pharmacol.* **11**, 600255 (2021). 33613279 10.3389/fphar.2020.600255 PMC7892439

[11] Senba, M. *et al.* Investigation of the efficacy of an administration plan for Tazobactam/piperacillin (TAZ/PIPC) and the incidence of kidney and hepatic disorders. *Yakugaku Zasshi* **137**, 1277–1284 (2017). 28966268 10.1248/yakushi.17-00016

[12] Kawanami, T. *et al.* Efficacy and safety of meropenem (3 g daily) in Japanese patients with refractory respiratory infections. *J. Infect. Chemother.* **20**, 768–773 (2014). 25193038 10.1016/j.jiac.2014.08.011

[13] David, S. & Hamilton, J. P. Drug-induced liver injury. *US Gastroenterol. Hepatol. Rev.* **6**, 73–80 (2010). 21874146 PMC3160634

[14] National Institute of Diabetes and Digestive and Kidney Diseases. *LiverTox: Clinical and Research Information on Drug-Induced Liver Injury*. https://pubmed.ncbi.nlm.nih.gov/31643176/ (Accessed 12 October 2023) (2019). 31643176

[15] McDonald, C. *et al.* Is high-dose β-lactam therapy associated with excessive drug toxicity in critically ill patients? *Minerva Anestesiol.* **82**, 957–965 (2016). 27054905

[16] Leitner, J. M., Graninger, W. & Thalhammer, F. Hepatotoxicity of antibacterials: Pathomechanisms and clinical. *Infection* **38**, 3–11 (2010). 20107858 10.1007/s15010-009-9179-z

[17] Grat, M. *et al.* Profile of gut microbiota associated with the presence of hepatocellular cancer in patients with liver cirrhosis. *Transplant. Proc.* **48**, 1687–1691 (2016). 27496472 10.1016/j.transproceed.2016.01.077

[18] Xue, L. *et al.* Probiotics may delay the progression of nonalcoholic fatty liver disease by restoring4 the gut microbiota structure and improving intestinal endotoxemia. *Sci. Rep.* **7**, 45176 (2017). 28349964 10.1038/srep45176 PMC5368635

[19] Fu, L. *et al.* Antibiotics enhancing drug-induced liver injury assessed for causality using roussel uclaf causality assessment method: emerging role of gut microbiota dysbiosis. *Front. Med.* **9**, 972518 (2022). 10.3389/fmed.2022.972518 PMC9500153 36160154

[20] Wang, J. *et al.* Gut microbial dysbiosis is associated with altered hepatic functions and serum metabolites in chronic hepatitis b patients. *Front. Microbiol.* **8**, 2222 (2017). 29180991 10.3389/fmicb.2017.02222 PMC5693892

[21] Luo, X. *et al.* Hepatic dysfunction induced by intestinal dysbacteriosis mainly manifests as immunologic abnormity in mice. *Pathog. Dis.* **78**, 041 (2020). 10.1093/femspd/ftaa041 32821930

[22] Zhang, H. L. *et al.* Profound impact of gut homeostasis on chemically-induced pro-tumorigenic inflammation and hepatocarcinogenesis in rats. *J. Hepatol.* **57**, 803–812 (2012). 22727732 10.1016/j.jhep.2012.06.011

[23] Kollef, M. H. Optimizing antibiotic therapy in the intensive care unit setting. *Crit. Care* **5**, 189–195 (2001). 11511331 10.1186/cc1022 PMC137278

[24] Tabah, A. *et al.* A systematic review of the definitions, determinants, and clinical outcomes of antimicrobial de-escalation in the intensive care unit. *Clin. Infect. Dis.* **62**, 1009–1017 (2016). 26703860 10.1093/cid/civ1199

[25] Lucena, M. I. *et al.* Determinants of the clinical expression of amoxicillin-clavulanate hepatotoxicity: A prospective series from Spain. *Hepatology* **44**, 850–856 (2006). 17006920 10.1002/hep.21324

[26] Singh, N., Rogers, P., Atwood, C. W., Wagener, M. M. & Yu, V. L. Short-course empiric antibiotic therapy for patients with pulmonary infiltrates in the intensive care unit: A proposed solution for indiscriminate antibiotic prescription. *Am. J. Respir. Crit. Care Med.* **162**, 505–511 (2000). 10934078 10.1164/ajrccm.162.2.9909095

[27] Dunbar, L. M. *et al.* Efficacy of 750-mg, 5-day levofloxacin in the treatment of community-acquired pneumonia caused by atypical pathogens. *Curr. Med. Res. Opin.* **20**, 555–563 (2004). 15119993 10.1185/030079904125003304

[28] Zhao, X. *et al.* A randomized controlled clinical trial of levofloxacin 750 mg versus 500 mg intravenous infusion in the treatment of community-acquired pneumonia. *Diagn. Microbiol. Infect. Dis.* **80**, 141–147 (2014). 25130297 10.1016/j.diagmicrobio.2013.11.008

[29] Pakistan Multicentre Amoxycillin Short Course Therapy (MASCOT) Pneumonia Study Group. Clinical efficacy of 3 days versus 5 days of oral amoxicillin for treatment of childhood pneumonia: A multicentre double-blind trial. *Lancet* **360**, 835–841 (2002). 12243918 10.1016/S0140-6736(02)09994-4

[30] Greenberg, D. *et al.* Short-course antibiotic treatment for community-acquired alveolar pneumonia in ambulatory children: A double-blind, randomized, placebo-controlled trial. *Pediatr. Infect. Dis. J.* **33**, 136–142 (2014). 23989106 10.1097/INF.0000000000000023

[31] El Moussaoui, R. *et al.* Effectiveness of discontinuing antibiotic treatment after three days versus eight days in mild to moderate-severe community acquired pneumonia: Randomised, double blind study. *Br. Med. J.* **332**, 1355 (2006). 16763247 10.1136/bmj.332.7554.1355 PMC1479094

[32] Uranga, A. *et al.* Duration of antibiotic treatment in community-acquired pneumonia: A multicenter randomized clinical trial. *JAMA Intern. Med.* **176**, 1257–1265 (2016). 27455166 10.1001/jamainternmed.2016.3633

[33] Dinh, A. *et al.* Honey, I shrunk the antibiotic therapy. *Clin. Infect. Dis.* **66**, 1981–1982 (2018). 29370382 10.1093/cid/ciy047

[34] Harris, J. A., Kolokathis, A., Campbell, M., Cassell, G. H. & Hammerschlag, M. R. Safety and efficacy of azithromycin in the treatment of community-acquired pneumonia in children. *Pediatr. Infect. Dis. J.* **17**, 865–871 (1998). 9802626 10.1097/00006454-199810000-00004

[35] Chastre, J. *et al.* PneumA Trial Group Comparison of 8 vs 15 days of antibiotic therapy for ventilator-associated pneumonia in adults: A randomized trial. *J. Am. Med. Assoc.* **290**, 2588–2598 (2003). 10.1001/jama.290.19.2588 14625336

[36] Capellier, G. *et al.* Early-onset ventilator-associated pneumonia in adults randomized clinical trial: Comparison of 8 versus 15 days of antibiotic treatment. *PLoS ONE* **7**, e41290 (2012). 22952580 10.1371/journal.pone.0041290 PMC3432026

[37] Jernelius, H., Zbornik, J. & Bauer, C. A. One or three weeks’ treatment of acute pyelonephritis? A double-blind comparison, using a fixed combination of pivampicillin plus pivmecillinam. *Acta Med. Scand.* **223**, 469–477 (1988). 3287839 10.1111/j.0954-6820.1988.tb15899.x

[38] de Gier, R. *et al.* A sequential study of intravenous and oral fleroxacin for 7 or 14 days in the treatment of complicated urinary tract infections. *Int. J. Antimicrob. Agents* **6**, 27–30 (1995). 18611681 10.1016/0924-8579(95)00011-V

[39] Talan, D. A. *et al.* Comparison of ciprofloxacin (7 days) and trimethoprim-sulfamethoxazole (14 days) for acute uncomplicated pyelonephritis pyelonephritis in women: A randomized trial. *J. Am. Med. Assoc.* **283**, 1583–1590 (2000). 10.1001/jama.283.12.1583 10735395

[40] Sandberg, T. *et al.* Ciprofloxacin for 7 days versus 14 days in women with acute pyelonephritis: A randomised, open-label and double-blind, placebo-controlled, non-inferiority trial. *Lancet* **380**, 484–490 (2012). 22726802 10.1016/S0140-6736(12)60608-4

[41] Peterson, J., Kaul, S., Khashab, M., Fisher, A. C. & Kahn, J. B. A double-blind, randomized comparison of levofloxacin 750 mg once-daily for five days with ciprofloxacin 400/500 mg twice-daily for 10 days for the treatment of complicated urinary tract infections and acute pyelonephritis. *Urology* **71**, 17–22 (2008). 18242357 10.1016/j.urology.2007.09.002

[42] Klausner, H. A. *et al.* A trial of levofloxacin 750 mg once daily for 5 days versus ciprofloxacin 400 mg and/or 500 mg twice daily for 10 days in the treatment of acute pyelonephritis. *Curr. Med. Res. Opin.* **23**, 2637–2645 (2007). 17880755 10.1185/030079907X233340

[43] Sawyer, R. G. *et al.* Trial of short-course antimicrobial therapy for intraabdominal infection. *N. Engl. J. Med.* **372**, 1996–2005 (2015). 25992746 10.1056/NEJMoa1411162 PMC4469182

[44] Montravers, P. *et al.* Short-course antibiotic therapy for critically ill patients treated for postoperative intra-abdominal infection: The DURAPOP randomised clinical trial. *Intens. Care Med.* **44**, 300–310 (2018). 10.1007/s00134-018-5088-x 29484469

[45] Yahav, D. *et al.* Seven versus 14 days of antibiotic therapy for uncomplicated gram-negative bacteremia: A noninferiority randomized controlled trial. *Clin. Infect. Dis.* **69**, 1091–1098 (2019). 30535100 10.1093/cid/ciy1054

[46] El Moussaoui, R. *et al.* Short-course antibiotic treatment in acute exacerbations of chronic bronchitis and COPD: A meta-analysis of double-blind studies. *Thorax* **63**, 415–422 (2008). 18234905 10.1136/thx.2007.090613

[47] Hepburn, M. J. *et al.* Comparison of short-course (5 days) and standard (10 days) treatment for uncomplicated cellulitis. *Arch. Intern. Med.* **164**, 1669–1674 (2004). 15302637 10.1001/archinte.164.15.1669

[48] Prokocimer, P., De Anda, C., Fang, E., Mehra, P. & Das, A. Tedizolid phosphate vs linezolid for treatment of acute bacterial skin and skin structure infections: The ESTABLISH-1 randomized trial. *J. Am. Med. Assoc.* **309**, 559–569 (2013). 10.1001/jama.2013.241 23403680

[49] Moran, G. J. *et al.* Tedizolid for 6 days versus linezolid for 10 days for acute bacterial skin and skin-structure infections (ESTABLISH-2): A randomised, double-blind, phase 3, non-inferiority trial. *Lancet Infect. Dis.* **14**, 696–705 (2014). 24909499 10.1016/S1473-3099(14)70737-6

[50] Bernard, L. *et al.* Antibiotic treatment for 6 weeks versus 12 weeks in patients with pyogenic vertebral osteomyelitis: An open-label, non-inferiority, randomised, controlled trial. *Lancet* **385**, 875–882 (2015). 25468170 10.1016/S0140-6736(14)61233-2

[51] Aguilar-Guisado, M. *et al.* Optimisation of empirical antimicrobial therapy in patients with haematological malignancies and febrile neutropenia (how long study): An open-label, randomised, controlled phase 4 trial. *Lancet Haematol.* **4**, e573–e583 (2017). 29153975 10.1016/S2352-3026(17)30211-9

[52] Royer, S., DeMerle, K. M., Dickson, R. P. & Prescott, H. C. Shorter versus longer courses of antibiotics for infection in hospitalized patients: A systematic review and meta-analysis. *J. Hosp. Med.* **13**, 336–342 (2018). 29370318 10.12788/jhm.2905 PMC5945333

[53] Potula, R., Dadhania, V. & Truant, A. L. Automated blood culture testing: A retrospective study indicates that a three-day incubation period is sufficient. *Med. Lab. Observ.* **47**, 8–10 (2015). 26495590

[54] Dever, J. B. & Sheikh, M. Y. Review article: Spontaneous bacterial peritonitis-bacteriology, diagnosis, treatment, risk factors and prevention. *Aliment. Pharmacol. Ther.* **41**, 1116–1131 (2015). 25819304 10.1111/apt.13172

[55] Song, J. U. & Lee, J. The impact of antimicrobial de-escalation therapy in culture-negative pneumonia: A systematic review and meta-analysis. *Korean J. Intern. Med.* **38**, 704–713 (2023). 37586813 10.3904/kjim.2023.115 PMC10493446

[56] Garnacho-Montero, J. *et al.* De-escalation of empirical therapy is associated with lower mortality in patients with severe sepsis and septic shock. *Intens. Care Med.* **40**, 32–40 (2014). 10.1007/s00134-013-3077-7 24026297

[57] Rubinstein, E. Short antibiotic treatment courses or how short is short? *Int. J. Antimicrob. Agents* **30**, 76–79 (2007). 10.1016/j.ijantimicag.2007.06.017 17826038

[58] McAteer, J. *et al.* Defining the optimal duration of therapy for hospitalized patients with complicated urinary tract infections and associated bacteremia. *Clin. Infect. Dis.* **76**, 1604–1612 (2023). 36633559 10.1093/cid/ciad009 PMC10411929

[59] Kang, C. I. *et al.* Bloodstream infections caused by antibiotic-resistant gram-negative bacilli: Risk factors for mortality and impact of inappropriate initial antimicrobial therapy on outcome. *Antimicrob. Agents Chemother.* **49**, 760–766 (2005). 15673761 10.1128/AAC.49.2.760-766.2005 PMC547233

[60] Zahar, J. R. *et al.* Outcomes in severe sepsis and patients with septic shock: Pathogen species and infection sites are not associated with mortality. *Crit. Care Med.* **39**, 1886–1895 (2011). 21516036 10.1097/CCM.0b013e31821b827c

[61] Jiang, F. *et al.* Incidence and risk factors of anti-tuberculosis drug induced liver injury (DILI): Large cohort study involving 4652 Chinese adult tuberculosis patients. *Liver Int.* **41**, 1565–1575 (2021). 33866661 10.1111/liv.14896

[62] Tomich, L. G., Nunez, M. & Mendes-Correa, M. C. Drug-induced liver injury in hospitalized HIV patients: High incidence and association with drugs for tuberculosis. *Ann. Hepatol.* **14**, 888–894 (2015). 26436361 10.5604/16652681.1171778

[63] Travlos, G. S. *et al.* Frequency and relationships of clinical chemistry and liver and kidney histopathology findings in 13-week toxicity studies in rats. *Toxicology* **107**, 17–29 (1996). 8597028 10.1016/0300-483X(95)03197-N

[64] Luangmonkong, T. *et al.* Targeting oxidative stress for the treatment of liver fibrosis. *Rev. Physiol. Biochem. Pharmacol.* **175**, 71–102 (2018). 29728869 10.1007/112_2018_10

[65] Imani, S., Buscher, H., Marriott, D., Gentili, S. & Sandaradura, I. Too much of a good thing: A retrospective study of β-lactam concentration-toxicity relationships. *J. Antimicrob. Chemother.* **72**, 2891–2897 (2017). 29091190 10.1093/jac/dkx209

[66] Kong, L. Z. *et al.* Pathogenesis, early diagnosis, and therapeutic management of alcoholic liver disease. *Int. J. Mol. Sci.* **20**, 2712 (2019). 31159489 10.3390/ijms20112712 PMC6600448

[67] Aday, A. W., Mitchell, M. C. & Casey, L. C. Alcoholic hepatitis: Current trends in management. *Curr. Opin. Gastroenterol.* **33**, 142–148 (2017). 28282320 10.1097/MOG.0000000000000359

[68] Lucey, M. R., Mathurin, P. & Morgan, T. R. Alcoholic hepatitis. *N. Engl. J. Med.* **360**, 2758–2769 (2009). 19553649 10.1056/NEJMra0805786

[69] Erol, A. & Karpyak, V. M. Sex and gender-related differences in alcohol use and its consequences: Contemporary knowledge and future research considerations. *Drug Alcohol Depend.* **156**, 1–13 (2015). 26371405 10.1016/j.drugalcdep.2015.08.023

[70] de Lemos, A. S. *et al.* Amoxicillin-Clavulanate-induced liver injury. *Dig. Dis. Sci.* **61**, 2406–2416 (2016). 27003146 10.1007/s10620-016-4121-6 PMC4945382

[71] Craig, D. G. *et al.* The systemic inflammatory response syndrome and sequential organ failure assessment scores are effective triage markers following paracetamol (acetaminophen) overdose. *Aliment. Pharmacol. Ther.* **34**, 219–228 (2011). 21554357 10.1111/j.1365-2036.2011.04687.x

[72] Zhao, Y., Zang, B. & Wang, Q. Prolonged versus intermittent β-lactam infusion in sepsis: A systematic review and meta-analysis of randomized controlled trials. *Ann. Intens. Care* **14**, 30 (2024). 10.1186/s13613-024-01263-9 PMC10874917 38368588

[73] Koch, A. *et al.* Increased liver stiffness denotes hepatic dysfunction and mortality risk in critically ill non-cirrhotic patients at a medical ICU. *Crit. Care* **15**, R266 (2011). 22082207 10.1186/cc10543 PMC3388655

[74] Anzai, T., Takahashi, K., Watanabe, M., Mochizuki, M. & Murashima, A. Adverse event reports in patients taking psychiatric medication during pregnancy from spontaneous reports in Japan and the United States: An approach using latent class analysis. *BMC Psychiatry* **20**, 118 (2020). 32164630 10.1186/s12888-020-02525-z PMC7068895

[75] Food, Drug Administration HHS. International conference on harmonisation; E2B(R3) electronic transmission of individual case safety reports; data elements and message specification; appendix on backwards and forwards compatibility; availability. *Notice Fed. Regist.* **79**, 9908–9909 (2014). 24611208

[76] Poluzzi, E., Raschi, E., Piccinni, C. & Ponti, F. Data mining techniques in pharmacovigilance: Analysis of the publicly accessible FDA adverse event reporting system (AERS). *IntechOpen* **12**, 266–302 (2012).

[77] Liu, Z. *et al.* Prognostic accuracy of the serum lactate level, the SOFA score and the qSOFA score for mortality among adults with sepsis. *Scand. J. Trauma Resusc. Emerg. Med.* **27**, 51 (2019). 31039813 10.1186/s13049-019-0609-3 PMC6492372

[78] Yoshida, H. *et al.* Use of broad-spectrum antimicrobials for more than 72 h and the detection of multidrug-resistant bacteria in Japanese intensive care units: A multicenter retrospective cohort study. *Antimicrob. Resist. Infect. Control* **11**, 119 (2022). 36175948 10.1186/s13756-022-01146-3 PMC9520832

[79] Aujesky, D. & Fine, M. J. The pneumonia severity index: A decade after the initial derivation and validation. *Clin. Infect. Dis.* **47**, 133–139 (2008). 18986279 10.1086/591394

[80] Vincent, J. L. *et al.* The SOFA (sepsis-related organ failure assessment) score to describe organ dysfunction/failure. *Intens. Care Med.* **22**, 707–710 (1996). 10.1007/BF01709751 8844239

[81] Zhang, H. F. *et al.* Serum prealbumin improves the sensitivity of pneumonia severity index in predicting 30-day mortality of CAP Patients. *Clin. Lab.* **66**, 1 (2020). 10.7754/Clin.Lab.2019.190929 32390376

[82] Lat, I., Foster, D. R. & Erstad, B. Drug-induced acute liver failure and gastrointestinal complications. *Crit. Care Med.* **38**, 175–187 (2010). 20502172 10.1097/CCM.0b013e3181de0db2

[83] Navarro, V. J. & Senior, J. R. Drug-related hepatotoxicity. *N. Engl. J. Med.* **354**, 731–739 (2006). 16481640 10.1056/NEJMra052270

[84] Johnson, P. J. *et al.* Assessment of liver function in patients with hepatocellular carcinoma: A new evidence-based approach-the ALBI grade. *J. Clin. Oncol.* **33**, 550–558 (2015). 25512453 10.1200/JCO.2014.57.9151 PMC4322258

[85] Sterling, R. K. *et al.* Development of a simple noninvasive index to predict significant fibrosis in patients with HIV/HCV coinfection. *Hepatology* **43**, 1317–1325 (2006). 16729309 10.1002/hep.21178

[86] Matsuo, S. *et al.* Revised equations for estimated GFR from serum creatinine in Japan. *Am. J. Kidney Dis.* **53**, 982–992 (2009). 19339088 10.1053/j.ajkd.2008.12.034

[87] R Core Team. *R: A Language and Environment for Statistical Computing*. https://www.R-project.org/ (Accessed 19 December 2022) (R Foundation for Statistical Computing, 2022).

