# Supplementary material for: An investigation of broad-spectrum antibiotic-induced liver injury based on the FDA Adverse Event Reporting System and retrospective observational study
Source: Sci Rep. 2024 Aug 6;14:18221. doi: 10.1038/s41598-024-69279-6 (PMC11303562; doi:10.1038/s41598-024-69279-6)
Supplement: Supplementary file 1 — Supplementary Information. [file 41598_2024_69279_MOESM1_ESM.docx]

**SUPPLEMENTARY INFORMATION**

**An investigation of broad-spectrum antibiotic–induced liver injury based on the FDA Adverse Event Reporting System and retrospective observational study**

**Chihiro Shiraishi ^1,2^, Hideo Kato ^1,2^, Toru Ogura ^3^ & Takuya Iwamoto ^1,2^**

1 Department of Pharmacy, Mie University Hospital, Tsu 514-8507, Japan

2 Department of Clinical Pharmaceutics, Division of Clinical Medical Science, Mie University Graduate School of Medicine, Tsu 514-8507, Japan

3 Clinical Research Support Center, Mie University Hospital, Tsu 514-8507, Japan

This PDF file includes:

Supplementary 1, Sources of infection (retrospective observational study) (n = 210)

Supplementary 2, Blood culture identification (retrospective observational study) (n = 210)

Supplementary 3, Comparison of patient background by sex (retrospective observational study) (n = 210)

Supplementary 4, Patient characteristics with non-septic infection (retrospective observational study) (n = 311)

Supplementary 5, Univariate and multivariate logistic regression analysis of TZP or MEM of the patients with non-septic infection (retrospective observational study) (n = 311)

Supplementary 6, Univariate and multivariate logistic regression analysis divided TZP and MEM of the patients with non-septic infection (retrospective observational study) (n = 311)

Supplementary 7, R scripts

Supplementary 8, Common Terminology Criteria for Adverse Events: elevation of alanine aminotransferase

Supplementary 1. Sources of infection (retrospective observational study) (n = 210)

Multiple answers were allowed.

|  | n (%) |
| --- | --- |
| Lower respiratory tract and lung infections | 105 (50.0) |
| Sepsis, bacteraemia | 30 (14.3) |
| Cardiac infections | 15 (7.1) |
| Abdominal and gastrointestinal infections | 16 (7.6) |
| [Coronavirus infections](javascript:detail('HLT',%20'10084510');) | 11 (5.2) |
| Urinary tract infections | 9 (4.3) |
| [Pyrexia](javascript:detail('PT',%20'10037660');) | 8 (3.8) |
| Central nervous system and spinal infections | 4 (1.9) |
| Wound infection | 4 (1.9) |
| Skin structures and soft tissue infections | 7 (3.3) |
| [Hepatobiliary and spleen infections](javascript:detail('HLT',%20'10064462');) | 1 (0.5) |
| Vascular infections | 3 (1.4) |

Supplementary 2. Blood culture identification (retrospective observational study) (n = 210)

ESBL, Extended-spectrum beta-lactamase

Multiple answers were allowed.

|  | n (%) |
| --- | --- |
| *Streptococcus pneumoniae* | 45 (21.4) |
| *Candida albicans* | 34 (16.2) |
| *Corynebacterium* spp*.* | 31 (14.8) |
| *Staphylococcus* spp*.* | 24 (11.4) |
| *Methicillin*–*susceptible Staphylococcus aureus* | 19 (9.0) |
| *Pseudomonas aeruginosa* | 18 (8.6) |
| *Enterococcus faecium* | 16 (7.6) |
| *Coagulase negative staphylococci* | 15 (7.1) |
| *Candida glabrata* | 14 (6.7) |
| *Enterococcus faecalis* | 11 (5.2) |
| *Methicillin*–*resistant Staphylococcus aureus* | 11 (5.2) |
| *Neisseria* spp*.* | 11 (5.2) |
| *Klebsiella pneumoniae* | 13 (6.2) |
| *Stenotrophomonas maltophilia* | 8 (3.8) |
| *Haemophilus influenzae* | 7 (3.3) |
| *Escherichia coli* | 6 (2.9) |
| *Staphylococcus aureus* | 5 (2.4) |
| *Candida* spp*.* | 4 (1.9) |
| *Enterobacter aerogenes (AmpC β*–*lactamase+)* | 5 (2.4) |
| *Escherichia coli (ESBL+)* | 4 (1.9) |
| *Serratia marcescens* | 3 (1.4) |
| *Streptococcus dysgalactiae* | 3 (1.4) |
| *Acinetobacter* spp*.* | 2 (1.0) |
| *Candida tropicalis* | 2 (1.0) |
| *Eikenella corrodens* | 2 (1.0) |
| *Enterobacter aerogenes* | 2 (1.0) |
| *Enterobacter* spp*. (AmpC β*–*lactamase+)* | 2 (1.0) |
| *Streptococcus constellatus* | 2 (1.0) |
| *Acinetobacter* | 1 (0.5) |
| *Acinetobacter baumannii* | 1 (0.5) |
| *Aerococcus urinae* | 1 (0.5) |
| *Aeromonas* | 1 (0.5) |
| *Aspergillus niger* | 1 (0.5) |
| *Bacillus cereus* | 1 (0.5) |

Supplementary 3. Comparison of patient background by sex (retrospective observational study) (n = 210)

ALBI score = (log_10_ T–Bil × 0.66) + (−0.085× Alb) ^84^

FIB–4 score = age × AST/platelet × (ALT)^1/2 85^

eGFR [mL/min/1.73 m^2^] = 194 × serum creatinine^−1.094^ × age^−0.287^ (×0.739 if female) ^86^

Alb, serum albumin; ALBI, albumin–bilirubin; ALP, alkaline phosphatase; ALT, alanine aminotransferase; AST, aspartate aminotransferase; BMI, body mass index; BUN, blood urea nitrogen; CHDF, continuous hemodiafiltration; CAS, caspofungin; CRP, C–reactive protein; DAP, daptomycin; ECMO, extracorporeal membrane oxygenation; eGFR, estimated glomerular filtration rate; FIB–4, Fibrosis–4; HD, hemodialysis; LIN, linezolid; MFG, micafungin; MEM, meropenem; NSAIDs, Non–Steroidal Anti–Inflammatory Drugs; PSI, pneumonia severity index; TEI, teicoplanin; RIF, rifampicin; SOFA, sequential organ failure assessment; SXT, sulfamethoxazole/trimethoprim; T–Bil, total bilirubin; VAN, vancomycin

Chi–squared test or Mann–Whitney U test

^*^Data presented as median [interquartile range].

|  | Male (n = 145) | Female (n = 65) | *p*–value |
| --- | --- | --- | --- |
|  | n (%) | n (%) |  |
| Age^*^, years | 72.0 [66.0–78.0] | 73.0 [64.5–83.0] | 0.361 |
| Body weight^*^, kg | 59.7 [50.6–68.6] | 51.1 [38.9–63.5] | <0.001 |
| BMI^*^, kg/m^2^ | 22.2 [18.7–25.2] | 22.7 [18.1–26.0] | 0.857 |
| Administration period^*^, day | 8.0 [5.5–14.0] | 7.0 [5.0–11.0] | 0.071 |
| Administration period ≥ seven days | 101 (69.7) | 33 (50.8) | 0.008 |
| Alcohol drinking history | 55 (37.9) | 4 (6.2) | <0.001 |
| SOFA score^*^ | 7.0 [5.0–10.5] | 6.0 [3.5–9.5] | 0.055 |
| PSI^*^ | 5.0 [4.0–5.0] | 4.0 [4.0–5.0] | 0.084 |
| Alb^*^, g/dL | 2.9 [2.5–3.3] | 2.9 [2.5–3.5] | 0.786 |
| ALP^*^, U/L | 211.0 [134.0–321.0] | 253.0 [158.5–375.0] | 0.105 |
| ALT^*^, U/L | 21.0 [14.0–28.0] | 20.0 [15.0–30.0] | 0.705 |
| ALBI score^*^ | –1.7 [–2.0 – –1.3] | –1.6 [–2.0 – –1.3] | 0.818 |
| FIB–4 score^*^ | 2.7 [1.9–6.2] | 3.2 [2.2–7.0] | 0.381 |
| AST^*^, U/L | 31.0 [21.0–49.5] | 35.0 [27.0–46.0] | 0.162 |
| T–Bil^*^, mg/dL | 0.8 [0.5–1.6] | 0.9 [0.6–1.5] | 0.905 |
| BUN^*^, mg/dL | 28.5 [19.7–46.7] | 24.0 [13.5–46.4] | 0.186 |
| eGFR^*^, mL/min/1.73m^2^ | 49.3 [22.7–84.1] | 51.7 [23.7–92.9] | 0.463 |
| ECMO | 17 (11.7) | 7 (10.8) | 1.000 |
| HD | 13 (9.0) | 3 (4.6) | 0.401 |
| CHDF | 37 (25.5) | 13 (20.0) | 0.484 |
| CRP^*^, mg/dL | 10.6 [6.0–17.5] | 11.3 [5.5–23.3] | 0.730 |
| Eosinophil^*^, /μL | 30.0 [0.0–140.0] | 20.0 [0.0–125.0] | 0.965 |
| Dose per day^*^, g per day | – | – | – |
| Dose/kg per day^*^, g/kg per day | – | – | – |
| Macrolide | 2 (1.4) | 1 (1.5) | 1.000 |
| SXT | 7 (4.8) | 3 (4.6) | 1.000 |
| Carbamazepine | 3 (2.1) | 0 (0.0) | 0.554 |
| Valproic acid | 2 (1.4) | 1 (1.5) | 1.000 |
| Acetaminophen | 30 (20.7) | 17 (26.2) | 0.377 |
| Acetaminophen dose | 1000.0 [400.0–1000.0] | 400.0 [400.0–1000.0] | 0.116 |
| Amiodarone | 7 (4.8) | 3 (4.6) | 1.000 |
| Statin | 4 (2.8) | 4 (6.2) | 0.257 |
| Propofol | 16 (11.0) | 7 (10.8) | 1.000 |
| RIF | 0 (0.0) | 0 (0.0) | 1.000 |
| NSAIDs | 16 (11.0) | 4 (6.2) | 0.319 |
| Opioids | 84 (57.9) | 23 (35.4) | 0.003 |
| VAN | 40 (27.6) | 13 (20.0) | 0.303 |
| TEI | 6 (4.1) | 1 (1.5) | 0.440 |
| LIN | 16 (11.0) | 2 (3.1) | 0.065 |
| DAP | 9 (6.2) | 2 (3.1) | 0.509 |
| CAS | 1 (0.7) | 0 (0.0) | 1.000 |
| MFG | 1 (0.7) | 0 (0.0) | 1.000 |

Supplementary 4. Patient characteristics with non-septic infection (retrospective observational study) (n = 311)

ALBI score = (log_10_ T–Bil × 0.66) + (−0.085 × Alb) ^84^

FIB–4 score = age × AST/platelet × (ALT)^1/2^ ^85^

eGFR [mL/min/1.73 m^2^] = 194 × serum creatinine^−1.094^ × age^−0.287^ (×0.739 if female) ^86^

Alb, serum albumin; ALBI, albumin–bilirubin; ALP, alkaline phosphatase; ALT, alanine aminotransferase; AST, aspartate aminotransferase; BMI, body mass index; BUN, blood urea nitrogen; CHDF, continuous hemodiafiltration; CAS, caspofungin; CRP, C–reactive protein; DAP, daptomycin; ECMO, extracorporeal membrane oxygenation; eGFR, estimated glomerular filtration rate; FIB–4, Fibrosis–4; HD, hemodialysis; LIN, linezolid; MFG, micafungin; MEM, meropenem; NSAIDs, Non–Steroidal Anti–Inflammatory Drugs; PSI, pneumonia severity index; TEI, teicoplanin; RIF, rifampicin; SOFA, sequential organ failure assessment; SXT, sulfamethoxazole/trimethoprim; T–Bil, total bilirubin; VAN, vancomycin

Chi–squared test or Mann–Whitney U test

^*^Data are presented as median [interquartile range].

|  | All (n = 311) | TZP (n = 105) | MEM (n = 206) | *p*–value |
| --- | --- | --- | --- | --- |
|  | n (%) | n (%) | n (%) |  |
| Age^*^, years | 71.0 [61.0–78.0] | 74.0 [65.0–82.0] | 70.0 [60.8–78.0] | 0.060 |
| Male | 244 (78.5) | 79 (75.2) | 136 (66.0) | 0.119 |
| Body weight^*^, kg | 57.6 [47.4–68.2] | 55.6 [47.3–67.0] | 58.3 [47.4–69.4] | 0.588 |
| BMI^*^, kg/m^2^ | 22.1 [18.6–25.4] | 22.5 [18.4–25.2] | 22.1 [18.7–25.7] | 0.772 |
| Administration period^*^, day | 7.0 [5.0–11.0] | 8.0 [5.0–14.0] | 7.0 [5.0–11.0] | 0.286 |
| Administration period ≥ seven days | 188 (60.5) | 65 (61.9) | 123 (59.7) | 0.903 |
| Alcohol drinking history | 91 (29.3) | 30 (28.6) | 61 (29.6) | 0.284 |
| SOFA score^*^ | 7.0 [4.0–10.0] | 6.0 [4.0–10.0] | 7.0 [4.0–10.0] | 0.542 |
| PSI^*^ | 5.0 [4.0–5.0] | 5.0 [4.0–5.0] | 5.0 [4.0–5.0] | 0.598 |
| Alb^*^, g/dL | 2.8 [2.5–3.3] | 2.8 [2.5–3.3] | 2.9 [2.5–3.3] | 0.737 |
| ALP^*^, U/L | 229.5 [144.0–335.0] | 253.5 [178.9–382.5] | 231.0 [154.5–332.5] | 0.179 |
| ALT^*^, U/L | 20.0 [14.0–28.0] | 20.0 [14.0–29.5] | 20.0 [13.0–28.0] | 0.568 |
| ALBI score^*^ | -1.7 [-2.0– -1.3] | -1.6 [-1.9– -1.3] | -1.7 [-2.0– -1.4] | 0.404 |
| FIB–4 score^*^ | 3.1 [1.9–6.3] | 2.5 [1.8–5.1] | 3.2 [1.9–6.3] | 0.187 |
| AST^*^, U/L | 34.0 [22.5–49.0] | 34.0 [21.0–51.0] | 33.0 [22.8–44.5] | 0.983 |
| T–Bil^*^, mg/dL | 0.8 [0.5–1.5] | 0.9 [0.5–1.4] | 0.8 [0.5–1.5] | 0.771 |
| BUN^*^, mg/dL | 30.2 [18.7–48.0] | 23.3 [16.1–43.4] | 29.9 [18.2–47.3] | 0.094 |
| eGFR^*^, mL/min/1.73m^2^ | 46.0 [21.7–85.3] | 64.8 [30.9–112.0] | 46.1 [22.5–84.4] | 0.014 |
| ECMO | 27 (8.7) | 8 (7.6) | 19 (9.2) | 0.832 |
| HD | 25 (8.0) | 8 (7.6) | 17 (8.3) | 1.000 |
| CHDF | 65 (20.9) | 17 (16.2) | 48 (23.3) | 0.184 |
| CRP^*^, mg/dL | 10.9 [5.6–19.7] | 10.5 [5.9–16.4] | 10.2 [4.9–18.5] | 0.770 |
| Eosinophil^*^, /μL | 30.0 [0.0–140.0] | 10.0 [0.0–110.0] | 40.0 [10.0–135.0] | 0.015 |
| Dose per day^*^, g per day | – | 13.5 [9.0–13.5] | 2.0 [1.0–3.0] | – |
| Dose/kg per day^*^, g/kg per day | – | 0.2 [0.1–0.3] | 0.03 [0.02–0.05] | – |
| Macrolide | 4 (1.3) | 2 (1.9) | 2 (1.0) | 0.606 |
| SXT | 9 (2.9) | 1 (1.0) | 8 (3.9) | 0.282 |
| Carbamazepine | 1 (0.3) | 0 (0.0) | 1 (0.5) | 1.000 |
| Valproic acid | 4 (1.3) | 2 (1.9) | 2 (1.0) | 0.606 |
| Acetaminophen | 56 (18.0) | 27 (25.7) | 29 (14.1) | 0.019 |
| Acetaminophen dose | 500.0 [400.0–1000.0] | 1000.0 [400.0–1000.0] | 400.0 [400.0–1000.0] | 0.067 |
| Amiodarone | 14 (4.5) | 5 (4.8) | 9 (4.4) | 1.000 |
| Statin | 9 (2.9) | 9 (8.6) | 0 (0.0) | <0.001 |
| Propofol | 39 (12.5) | 13 (12.4) | 26 (12.6) | 1.000 |
| RIF | 0 (0.0) | 0 (0.0) | 0 (0.0) | 1.000 |
| NSAIDs | 27 (8.7) | 10 (9.5) | 17 (8.3) | 0.677 |
| Opioids | 147 (47.3) | 57 (54.2) | 90 (43.7) | 0.093 |
| VAN | 73 (23.5) | 22 (21.0) | 51 (24.8) | 0.274 |
| TEI | 11 (3.5) | 4 (3.8) | 7 (3.4) | 1.000 |
| LIN | 30 (9.6) | 8 (7.6) | 22 (10.7) | 0.258 |
| DAP | 17 (5.5) | 4 (3.8) | 13 (6.3) | 0.262 |
| CAS | 1 (0.3) | 1 (1.0) | 0 (0.0) | 0.338 |
| MFG | 3 (1.0) | 1 (1.0) | 2 (1.0) | 1.000 |

Supplementary 5. Univariate and multivariate logistic regression analysis of TZP or MEM of the patients with non-septic infection (retrospective observational study) (n = 311)

Adjusted coefficient of determination = 0.104 (*p* < 0.001)

ALBI score = (log_10_ T–Bil × 0.66) + (−0.085 × Alb) ^84^

FIB–4 score = age × AST/platelet × (ALT)^1/2^ ^85^

eGFR [mL/min/1.73 m^2^] = 194 × serum creatinine^−1.094^ × age^−0.287^ (×0.739 if female) ^86^

Administration period ≥ seven days was coded 1, and < seven days was coded 0, and univariate and multivariate binary logistic regression analysis was conducted.

Variables with *p* < 0.05 were included in the multivariate model. Using the stepwise forward selection method, potential independent variables were further examined to construct the final model.

In the multivariate model, all independent variables were considered as candidates. The final model was constructed using the stepwise forward selection method, with a selection criterion of *p* < 0.05. Selected variables are presented as OR [95% CI] and *p*-value, while unselected variables are presented as "–".

Alb, serum albumin; ALBI, albumin–bilirubin; ALP, alkaline phosphatase; ALT, alanine aminotransferase; AST, aspartate aminotransferase; BMI, body mass index; BUN, blood urea nitrogen; CI, confidence interval; CRP, C–reactive protein; eGFR, estimated glomerular filtration rate; FIB–4, Fibrosis–4; MEM, meropenem; OR, odds ratio; PSI, pneumonia severity index; SOFA, sequential organ failure assessment; T–Bil, total bilirubin; TZP, tazobactam/piperacillin

|  | TZP or MEM (n = 311) | | | |  |  |
| --- | --- | --- | --- | --- | --- | --- |
|  | Univariate analysis | | Multivariate analysis  (variable selection) | | | |
|  | OR [95% CI] | *p*–value | OR [95% CI] | *p*–value | |  |
| Age, years | 0.318 [0.108–0.938] | 0.037 | – | – | |  |
| Male | 1.775 [1.115–2.824] | 0.014 | – | – | |  |
| Body weight, kg | 1.015 [1.003–1.028] | 0.012 | – | – | |  |
| BMI, kg/m^2^ | 1.019 [0.984–1.056] | 0.289 | – | – | |  |
| Administration period ≥ seven days | 3.977 [2.394–6.777] | <0.001 | 4.501 [2.659–7.858] | <0.001 | |  |
| Alcohol drinking history | 0.862 [0.531–1.399] | 0.548 | – | – | |  |
| SOFA score | 1.777 [1.357–1.881] | 0.011 | – | – | |  |
| PSI | 0.788 [0.644–0.964] | 0.011 | – | – | |  |
| Alb, g/dL | 0.788 [0.172–3.604] | 0.758 | – | – | |  |
| ALP, U/L | 1.000 [0.999–1.001] | 0.619 | – | – | |  |
| ALT, U/L | 12.488 [2.960–52.675] | 0.293 | – | – | |  |
| ALBI score | 1.142 [0.751–1.737] | 0.534 | – | – | |  |
| FIB–4 score | 0.460 [0.049–4.307] | 0.484 | – | – | |  |
| AST, U/L | 3.155 [0.749–13.287] | 0.116 | – | – | |  |
| T–Bil, mg/dL | 0.488 [0.009–26.720] | 0.714 | – | – | |  |
| BUN, mg/dL | 0.687 [0.116–4.076] | 0.678 | – | – | |  |
| eGFR, mL/min/1.73m^2^ | 1.005 [1.000–1.009] | 0.045 | – | – | |  |
| CRP, mg/dL | 1.005 [0.984–1.027] | 0.638 | – | – | |  |
| Eosinophil, /μL | 0.998 [0.996–1.000] | 0.038 | – | – | |  |
| Co–administered drugs | 0.938 [0.575–1.529] | 0.796 | – | – | |  |
| Acetaminophen dose, mg per day | 1.000 [0.999–1.001] | 0.532 | – | – | |  |
| MEM | 0.860 [0.549–1.347] | 0.511 | – | – | |  |

Supplementary 6. Univariate and multivariate logistic regression analysis divided TZP and MEM of the patients with non-septic infection (retrospective observational study) (n =311)

TZP; adjusted coefficient of determination = 0.148 (*p* < 0.001)

MEM; adjusted coefficient of determination = 0.129 (*p* < 0.001)

ALBI score = (log_10_ T–Bil × 0.66) + (−0.085× Alb) ^84^

FIB–4 score = age × AST/platelet × (ALT)^1/2^ ^85^

eGFR [mL/min/1.73 m^2^] = 194 × serum creatinine^−1.094^ × age^−0.287^ (×0.739 if female) ^86^

Administration period ≥ seven days was coded 1, and < seven days was coded 0, and univariate and multivariate binary logistic regression analysis was conducted.

Variables with *p* < 0.05 were included in the multivariate model. Using the stepwise forward selection method, potential independent variables were further examined to construct the final model.

In the multivariate model, all independent variables were considered as candidates. The final model was constructed using the stepwise forward selection ７method, with a selection criterion of *p* < 0.05. Selected variables are presented as OR [95% CI] and *p*-value, while unselected variables are presented as "–".

Alb, serum albumin; ALBI, albumin–bilirubin; ALP, alkaline phosphatase; ALT, alanine aminotransferase; AST, aspartate aminotransferase; BMI, body mass index; BUN, blood urea nitrogen; CI, confidence interval; CRP, C–reactive protein; eGFR, estimated glomerular filtration rate; FIB–4, Fibrosis–4; MEM, meropenem; OR, odds ratio; PSI, pneumonia severity index; SOFA, sequential organ failure assessment; T–Bil, total bilirubin; TZP, tazobactam/piperacillin

|  | TZP (n = 105) | | | |  | MEM (n = 206) | | | |
| --- | --- | --- | --- | --- | --- | --- | --- | --- | --- |
|  | Univariate analysis | | Multivariate analysis  (variable selection) | |  | Univariate analysis | | Multivariate analysis  (variable selection) | |
|  | OR [95% CI] | *p*–value | OR [95% CI] | *p*–value |  | OR [95% CI] | *p*–value | OR [95% CI] | *p*–value |
| Age, years | 0.989 [0.966–1.014] | 0.394 | – | – |  | 0.983 [0.967–0.999] | 0.041 | – | – |
| Male | 1.200 [0.521–2.763] | 0.667 | – | – |  | 2.025 [1.090–3.763] | 0.023 | 2.365 [1.208–4.797] | 0.014 |
| Body weight, kg | 1.013 [0.992–1.034] | 0.199 | – | – |  | 1.017 [1.001–1.031] | 0.029 | – | – |
| BMI, kg/m^2^ | 1.033 [0.973–1.096] | 0.264 | – | – |  | 1.010 [0.965–1.058] | 0.662 | – | – |
| Administration period ≥ seven days | 4.813 [2.003–12.659] | 0.001 | 4.170 [1.676–11.265] | 0.002 |  | 3.614 [1.952–6.932] | <0.001 | 4.360 [2.246–8.887] | <0.001 |
| Alcohol drinking history | 0.707 [0.293–1.704] | 0.435 | – | – |  | 0.899 [0.499–1.621] | 0.724 | – | – |
| SOFA score | 1.189 [1.070–1.321] | <0.001 | – | – |  | 1.053 [0.989–1.122] | 0.107 | – | – |
| PSI | 0.871 [0.608–1.248] | 0.452 | – | – |  | 0.749 [0.586–0.958] | 0.012 | – | – |
| Alb, g/dL | 0.636 [0.303–1.333] | 0.224 | – | – |  | 1.106 [0.695–1.762] | 0.670 | – | – |
| ALP, U/L | 1.000 [0.999–1.001] | 0.925 | – | – |  | 0.999 [0.997–1.000] | 0.042 | – | – |
| ALT, U/L | 1.063 [1.021–1.108] | 0.002 | 1.064 [1.019–1.115] | 0.004 |  | 1.029 [1.000–1.058] | 0.046 | – | – |
| ALBI score | 2.013 [0.891–4.551] | 0.083 | – | – |  | 0.896 [0.542–1.483] | 0.670 | – | – |
| FIB–4 score | 1.044 [0.975–1.118] | 0.192 | – | – |  | 0.981 [0.950–1.013] | 0.199 | – | – |
| AST, U/L | 1.020 [1.004–1.037] | 0.009 | – | – |  | 1.001 [0.992–1.010] | 0.809 | – | – |
| T–Bil, mg/dL | 1.100 [0.964–1.255] | 0.109 | – | – |  | 0.968 [0.891–1.052] | 0.286 | – | – |
| BUN, mg/dL | 1.012 [0.998–1.026] | 0.090 | – | – |  | 0.992 [0.982–1.002] | 0.107 | – | – |
| eGFR, mL/min/1.73m^2^ | 0.997 [0.989–1.004] | 0.412 | – | – |  | 1.009 [1.003–1.015] | 0.002 | 1.012 [1.006–1.020] | <0.001 |
| CRP, mg/dL | 0.992 [0.945–1.041] | 0.732 | – | – |  | 1.010 [0.985–1.034] | 0.441 | – | – |
| Eosinophil, /μL | 0.996 [0.992–1.001] | 0.040 | – | – |  | 0.999 [0.997–1.001] | 0.223 | – | – |
| Dose per day, g per day | 1.009 [0.908–1.120] | 0.872 | – | – |  | 1.040 [1.001–1.091] | 0.003 | – | – |
| Dose/kg per day, g/kg per day | 0.157 [0.003–8.302] | 0.355 | – | – |  | 1.828 [0.309–10.805] | 0.507 | – | – |
| Co–administered drugs | 0.535 [0.233–1.230] | 0.013 | – | – |  | 1.284 [0.697–2.366] | 0.424 | – | – |
| Acetaminophen dose, mg per day | 1.000 [0.998–1.001] | 0.924 | – | – |  | 1.000 [0.997–1.001] | 0.316 | – | – |

Supplementary 7. R scripts

#The R script can be executed after unzipping "faers_ascii_yyyyQq.zip" (yyyy and q present the year and

#quarter, respectively, from 2013Q1 to 2022Q3.) of "ASCII" to "C:\\".

install.packages("bit64"); install.packages("data.table"); install.packages("lubridate")

install.packages("Epi"); install.packages("dplyr")

library(data.table); library(lubridate); library(Epi); library(dplyr)

folder<-"C:\\FAERS"; "%not.in%"<-Negate("%in%")

start_y<-2013; start_q<-1; end_y<-2022; end_q<-3

case0<-c(10521863,11114009,11982392,14973456,14993574,15061633,16337820,

16726751,16931136,16931917,18596756)

n_of_drugs<-12

drug.name1<-c("CEFEPIME","MAXIPIME","AXEPIM")

drug.name2<-c("DORIPENEM","FINIBAX","DORIBAX","DORIPREX")

drug.name3<-c("BIAPENEM","OMEGACIN")

drug.name4<-c("MEROPENEM","MERONEM","MEROPEN","MERREM","MEPEM")

drug.name5<-c("CEFTOLOZANE","ZERBAXA")

drug.name5z<-c("TAZOBACTAM")

drug.name6<-c("PIPERACILLIN","ZOSYN")

drug.name6z<-c("TAZOBACTAM")

drug.name7<-c("GARENOXACIN","GARENOXACIN")

drug.name8<-c("SITAFLOXACIN","GRACEVIT")

drug.name9<-c("CIPROFLOXACIN","CIPRO","CIPRODEX","CIPROXIN", "CIFLOX","CIPROBAY")

drug.name10<-c("TOSUFLOXACIN","OZEX")

drug.name11<-c("MOXIFLOXACIN","AVELOX","VIGAMOX","AVALOX","IZILOX", "VEGAMOX","MOXEZA")

drug.name12<-c("LEVOFLOXACIN","LEVAQUIN","CRAVIT","TAVANIC")

AE.name1<-"Drug-induced liver injury"

indi_pt.name1<-"Sepsis"; indi_pt.name2<-"Disseminated intravascular coagulation"

drug.name_all<-NULL

for(i in 1:n_of_drugs){

eval(parse(text=paste("drug.name",i,"a<-paste(substr(drug.name", i,"[1],1,1),

tolower(substr(drug.name",i,"[1],2,nchar(drug.name", i,"[1]))),sep='')",sep="")))

eval(parse(text=paste("drug.name_all<-c(drug.name_all,drug.name",i,"a)",sep="")))

eval(parse(text=paste("DRUG_",i,"_all<-NULL",sep="")))

}

DRUG_all<-NULL; DEMO_all<-NULL; THER_all<-NULL; REAC_all<-NULL; INDI_all<-NULL

if(start_y==end_y){

qq<-start_q:end_q

}

if(start_y<end_y){

qq<-c(start_q:4,rep(1:4,end_y-start_y-1),1:end_q)

}

if(start_y==end_y){

yy<-rep(start_y,end_q-start_q+1)

}

if(start_y<end_y){

yy<-rep(start_y,length(start_q:4))

if((start_y+1)<=(end_y-1)){

for(i in (start_y+1):(end_y-1)){

yy<-c(yy,rep(i,4))

}

}

yy<-c(yy,rep(end_y,length(1:end_q)))

}

for(i in 1:length(yy)){

DRUG<-fread(paste(folder,"\\faers_ascii_",yy[i],"Q",qq[i],"\\ASCII\\DRUG",

yy[i]%%100,"Q",qq[i],".txt",sep=""),sep="$",quote="")

if(yy[i]==2018&qq[i]==1){

DEMO<-fread(paste(folder,"\\faers_ascii_",yy[i],"Q",qq[i],"\\ASCII\\DEMO",

yy[i]%%100,"Q",qq[i],"_new.txt",sep=""),sep="$")

}else{

DEMO<-fread(paste(folder,"\\faers_ascii_",yy[i],"Q",qq[i],"\\ASCII\\DEMO",

yy[i]%%100,"Q",qq[i],".txt",sep=""),sep="$")

}

THER<-fread(paste(folder,"\\faers_ascii_",yy[i],"Q",qq[i],"\\ASCII\\THER",

yy[i]%%100,"Q",qq[i],".txt",sep=""),sep="$")

REAC<-fread(paste(folder,"\\faers_ascii_",yy[i],"Q",qq[i],"\\ASCII\\REAC",

yy[i]%%100,"Q",qq[i],".txt",sep=""),sep="$")

INDI<-fread(paste(folder,"\\faers_ascii_",yy[i],"Q",qq[i],"\\ASCII\\INDI",

yy[i]%%100,"Q",qq[i],".txt",sep=""),sep="$")

names(DRUG)[names(DRUG)=="lot_nbr"]<-"lot_num"

DRUG$nda_num<-as.character(DRUG$nda_num)

if(sum(names(DRUG)=="prod_ai")==0){

DRUG$prod_ai<-NA

}

names(DEMO)[names(DEMO)=="gndr_cod"]<-"sex"

if(sum(names(DEMO)=="age_grp")==0){

DEMO$age_grp<-NA

}

if(sum(names(DEMO)=="lit_ref")==0){

DEMO$lit_ref<-NA

}

if(sum(names(DEMO)=="auth_num")==0){

DEMO$auth_num<-NA

}

if(sum(names(REAC)=="drug_rec_act")==0){

REAC$drug_rec_act<-NA

}

drugname<-gsub("[.]+$","",DRUG$drugname)

DRUG<-cbind(DRUG,DRUGNAME=toupper(drugname))

DRUG$type<-0

DRUG_f1<-function(drug.name0){

rbind(

DRUG[grep(paste("^",drug.name0,sep=""),DRUG$prod_ai),],

DRUG[stringr::str_detect(DRUG$prod_ai,pattern=paste("\\\\",drug.name0,sep="")),]

)

}

for(k in 1:n_of_drugs){

eval(parse(text=paste("DRUG_",k,"<-DRUG_f1(drug.name",k,"[1])",sep="")))

}

DRUG_5<-rbind(

DRUG_5[grep(paste("^",drug.name5z[1],sep=""),DRUG_5$prod_ai),],

DRUG_5[stringr::str_detect(DRUG_5$prod_ai,pattern=paste("\\\\",drug.name5z[1],sep="")),]

)

DRUG_6<-rbind(

DRUG_6[grep(paste("^",drug.name6z[1],sep=""),DRUG_6$prod_ai),],

DRUG_6[stringr::str_detect(DRUG_6$prod_ai,pattern=paste("\\\\",drug.name6z[1],sep="")),]

)

DRUG2<-NULL

for(k in 1:n_of_drugs){

DRUG1<-subset(DRUG,is.na(DRUG$prod_ai))

if(k %not.in% c(5,6)){

eval(parse(text=paste("for(j in 1:length(drug.name",k,")){

DRUG_",k,"<-rbind(DRUG_",k,",DRUG1%>%filter(grepl(drug.name",k,"[j],DRUGNAME)))

delkey<-DRUG_",k,"[,c('primaryid','caseid','drug_seq')]

delkey$delkey<-1

DRUG1<-merge(DRUG1,delkey,by=c('primaryid','caseid','drug_seq'),all=T,allow.cartesian=T)

DRUG1<-DRUG1[is.na(DRUG1$delkey),-'delkey']

}",sep="")))

eval(parse(text=paste("DRUG_",k,"$type<-",k,sep="")))

eval(parse(text=paste("DRUG2<-rbind(DRUG2,DRUG_",k,")",sep="")))

}

}

DRUG_5y<-rbind(

DRUG1[grep(paste("^",drug.name5[1],sep=""),DRUG1$DRUGNAME),],

DRUG1[stringr::str_detect(DRUG1$DRUGNAME,pattern=paste("\\\\",drug.name5[1],sep="")),]

)

DRUG_5y<-rbind(

DRUG_5y[grep(paste("^",drug.name5z[1],sep=""),DRUG_5y$DRUGNAME),],

DRUG_5y[stringr::str_detect(DRUG_5y$DRUGNAME,pattern=paste("\\\\",drug.name5z[1],sep="")),]

)

DRUG_5z<-rbind(

DRUG1[grep(paste("^",drug.name5[2],sep=""),DRUG1$DRUGNAME),],

DRUG1[stringr::str_detect(DRUG1$DRUGNAME,pattern=paste("\\\\",drug.name5[2],sep="")),]

)

DRUG_5<-rbind(DRUG_5,DRUG_5y,DRUG_5z)

DRUG_5$type<-5

DRUG2<-rbind(DRUG2,DRUG_5)

DRUG_6y<-rbind(

DRUG1[grep(paste("^",drug.name6[1],sep=""),DRUG1$DRUGNAME),],

DRUG1[stringr::str_detect(DRUG1$DRUGNAME,pattern=paste("\\\\",drug.name6[1],sep="")),]

)

DRUG_6y<-rbind(

DRUG_6y[grep(paste("^",drug.name6z[1],sep=""),DRUG_6y$DRUGNAME),],

DRUG_6y[stringr::str_detect(DRUG_6y$DRUGNAME,pattern=paste("\\\\",drug.name6z[1],sep="")),]

)

DRUG_6z<-rbind(

DRUG1[grep(paste("^",drug.name6[2],sep=""),DRUG1$DRUGNAME),],

DRUG1[stringr::str_detect(DRUG1$DRUGNAME,pattern=paste("\\\\",drug.name6[2],sep="")),]

)

DRUG_6<-rbind(DRUG_6,DRUG_6y,DRUG_6z)

DRUG_6$type<-6

DRUG2<-rbind(DRUG2,DRUG_6)

DRUG2<-rbind(DRUG2,DRUG[DRUG$caseid %in% case0,])

key0<-DRUG2[,c('primaryid','caseid')]

key0$key<-1

DEMO2<-merge(DEMO,key0,by=c('primaryid','caseid'),all=T,allow.cartesian=T)

DEMO2<-DEMO2[DEMO2$key==1,-'key']

REAC2<-merge(REAC,key0,by=c('primaryid','caseid'),all=T,allow.cartesian=T)

REAC2<-REAC2[REAC2$key==1,-'key']

key0<-DRUG2[,c('primaryid','caseid','drug_seq')]

key0$key<-1

THER2<-merge(THER,key0,by.x=c('primaryid','caseid','dsg_drug_seq'),

by.y=c('primaryid','caseid','drug_seq'),all=T,allow.cartesian=T)

THER2<-THER2[THER2$key==1,-'key']

INDI2<-merge(INDI,key0,by.x=c('primaryid','caseid','indi_drug_seq'),

by.y=c('primaryid','caseid','drug_seq'),all=T,allow.cartesian=T)

INDI2<-INDI2[INDI2$key==1,-'key']

DRUG_all<-rbind(DRUG_all,DRUG2)

DEMO_all<-rbind(DEMO_all,DEMO2)

THER_all<-rbind(THER_all,THER2)

REAC_all<-rbind(REAC_all,REAC2)

INDI_all<-rbind(INDI_all,INDI2)

}

N0<-length(unique(DRUG_all$primaryid))

DEMO_all<-DEMO_all[order(DEMO_all$caseid,DEMO_all$caseversion),]

DEMO2_all<-aggregate(DEMO_all,by=list(rleid(DEMO_all$caseid)),FUN=function(x)x[length(x)])[,c(names(DEMO_all))]

dat<-merge(DRUG_all,DEMO2_all,by=c("primaryid","caseid"),all.x=T,allow.cartesian=T)

dat<-merge(dat,THER_all, by.x=c("primaryid","caseid","drug_seq"),

by.y=c("primaryid","caseid","dsg_drug_seq"),all.x=T,allow.cartesian=T)

dat<-merge(dat,INDI_all, by.x=c("primaryid","caseid","drug_seq"),

by.y=c("primaryid","caseid","indi_drug_seq"),all.x=T,allow.cartesian=T)

dat<-dat[order(dat$caseid),]

dat<-subset(dat,is.na(dat$caseversion)==F)

dat<-subset(dat,dat$type>=1)

for(k in 1:n_of_drugs){

eval(parse(text=paste("dat$type",k,"<-0",sep="")))

eval(parse(text=paste("dat[dat$caseid %in% subset(dat,dat$type==",k,")$caseid,'type",k,"']<-1",sep="")))

}

dat$indi_pt1<-0

dat[dat$indi_pt==indi_pt.name1,"indi_pt1"]<-1

dat$indi_pt2<-0

dat[dat$indi_pt==indi_pt.name2,"indi_pt2"]<-1

tab<-rbind(table(dat$indi_pt1),table(dat$indi_pt2))

dat$indi_pt1a<-0

dat[dat$caseid %in% subset(dat,dat$indi_pt1==1)$caseid,"indi_pt1a"]<-1

dat$indi_pt2a<-0

dat[dat$caseid %in% subset(dat,dat$indi_pt2==1)$caseid,"indi_pt2a"]<-1

#Data-handling of patient background

dat$sex2<-factor(0,levels=c(0,1))

dat[dat$sex=="M","sex2"]<-factor(1,levels=c(0,1))

dat[dat$sex %not.in% c("F","M"),"sex2"]<-NA

dat$age2<-as.numeric(dat$age)

age0<-as.numeric(dat$age)

dat[dat$age_cod=="DEC","age2"]<-age0[which(dat$age_cod=="DEC")]*10+5

dat[dat$age_cod=="MON","age2"]<-floor(age0[which(dat$age_cod=="MON")]/12)

dat[dat$age_cod=="WK","age2"]<-floor(age0[which(dat$age_cod=="WK")]/(365.25/7))

dat[dat$age_cod=="DY","age2"]<-floor(age0[which(dat$age_cod=="DY")]/365.25)

dat[dat$age_cod=="HR","age2"]<-floor(age0[which(dat$age_cod=="HR")]/365.25/24)

dat$wt2<-as.numeric(sub(",","",dat$wt))

dat[dat$wt_cod=="LBS","wt2"]<-dat[dat$wt_cod=="LBS","wt2"]*0.454

dat$Cephem<-0

dat[dat$type1==1,"Cephem"]<-1

dat$Carbapenem<-0

dat[dat$type2==1|dat$type3==1|dat$type4==1,"Carbapenem"]<-1

dat$beta<-0

dat[dat$type5==1|dat$type6==1,"beta"]<-1

dat$Quinolone<-0

dat[dat$type7==1|dat$type8==1|dat$type9==1|dat$type10==1|dat$type11==1|dat$type12==1,"Quinolone"]<-1

N1<-length(unique(dat$caseid))

REAC_all1<-REAC_all[(REAC_all$primaryid %in% dat$primaryid & REAC_all$caseid %in% dat$caseid),]

REAC_all1$AE<-0

REAC_all1[REAC_all1$pt==AE.name1,"AE"]<-1

REAC_all2<-REAC_all1[order(REAC_all1$caseid,REAC_all1$AE),]

REAC_all2<-aggregate(REAC_all2,by=list(rleid(REAC_all2$caseid)),FUN=function(x)x[length(x)])[,c(names(REAC_all2))]

dat0<-aggregate(dat,by=list(rleid(dat$caseid)),FUN=function(x)x[1])[,c(names(dat))]

dat_PT<-merge(dat0,REAC_all2,by=c("primaryid","caseid"),all.x=T,allow.cartesian=T)

rawdata<-dat_PT[c("primaryid","caseid","age","age_cod","age2","sex","sex2", "wt","wt_cod","wt2","event_dt","mfr_dt",

"init_fda_dt","fda_dt","rept_cod","rept_dt","AE","occp_cod","reporter_country","occr_country")]

rawdata$FEP<-dat_PT$type1

rawdata$DOR<-dat_PT$type2

rawdata$BIPM<-dat_PT$type3

rawdata$MEM<-dat_PT$type4

rawdata$C_T<-dat_PT$type5

rawdata$TZP<-dat_PT$type6

rawdata$GRNX<-dat_PT$type7

rawdata$STFX<-dat_PT$type8

rawdata$CIP<-dat_PT$type9

rawdata$TFLX<-dat_PT$type10

rawdata$MXF<-dat_PT$type11

rawdata$LVX<-dat_PT$type12

rawdata$Sepsis<-dat_PT$indi_pt1a

#Table 1

NN<-table(dat_PT$AE)

f_tab<-function(dat0,vari){

tab1<-table(dat0,vari,useNA="ifany")

tab2<-NULL

for(k in 1:ncol(tab1)){

per<-tab1[,k]/NN[k]*100

vec<-paste(gsub(" ","",format(tab1[,k],big.mark=","))," (",gsub(" ","",format(round(per,1),nsmall=1)),")",sep="")

tab2<-cbind(tab2,vec)

}

rownames(tab2)<-rownames(tab1)

colnames(tab2)<-colnames(tab1)

return(tab2)

}

f_summary<-function(dat0,vari){

summary0<-tapply(vari,dat0,summary)

summary1<-as.matrix(bind_rows(summary0))

summary1[is.na(summary1[,"NA's"]),"NA's"]<-0

summary2<-NULL

for(k in 1:nrow(summary1)){

summary2<-cbind(summary2,c(paste(format(round(summary1[k,3],1),nsmall=1), " [",format(round(

summary1[k,2],1),nsmall=1),"-",format(round(summary1[k,5],1),nsmall=1),"]",sep=""),

paste(format(summary1[k,7],big.mark=",")," (",format(round(summary1[k,7]/NN[k]*100,1),nsmall=1),")",sep="")))

}

return(summary2)

}

Table1<-rbind(paste("(n = ",gsub(" ","",format(NN,big.mark=",")),")",sep=""),Sex="",f_tab(dat_PT$sex2,dat_PT$AE))

Table1<-rbind(Table1,f_summary(dat_PT$AE,dat_PT$age2))

Table1<-rbind(Table1,f_tab(dat_PT$Cephem,dat_PT$AE)[2,])

Table1<-rbind(Table1,f_tab(dat_PT$type1,dat_PT$AE)[2,])

Table1<-rbind(Table1,f_tab(dat_PT$Carbapenem,dat_PT$AE)[2,])

Table1<-rbind(Table1,f_tab(dat_PT$type2,dat_PT$AE)[2,])

Table1<-rbind(Table1,f_tab(dat_PT$type3,dat_PT$AE)[2,])

Table1<-rbind(Table1,f_tab(dat_PT$type4,dat_PT$AE)[2,])

Table1<-rbind(Table1,f_tab(dat_PT$beta,dat_PT$AE)[2,])

Table1<-rbind(Table1,f_tab(dat_PT$type5,dat_PT$AE)[2,])

Table1<-rbind(Table1,f_tab(dat_PT$type6,dat_PT$AE)[2,])

Table1<-rbind(Table1,f_tab(dat_PT$Quinolone,dat_PT$AE)[2,])

Table1<-rbind(Table1,f_tab(dat_PT$type7,dat_PT$AE)[2,])

Table1<-rbind(Table1,f_tab(dat_PT$type8,dat_PT$AE)[2,])

Table1<-rbind(Table1,f_tab(dat_PT$type9,dat_PT$AE)[2,])

Table1<-rbind(Table1,f_tab(dat_PT$type10,dat_PT$AE)[2,])

Table1<-rbind(Table1,f_tab(dat_PT$type11,dat_PT$AE)[2,])

Table1<-rbind(Table1,f_tab(dat_PT$type12,dat_PT$AE)[2,])

Table1<-rbind(Table1,f_tab(dat_PT$indi_pt1a,dat_PT$AE)[2,])

rownames(Table1)<-c("","Sex"," Female"," Male"," Unknown","Age, years"," Unknown",

"The fourth–generation cephalosporin"," FEP","Carbapenem"," DOR"," BIPM"," MEM",

"β-lactam and β-lactamase inhibitor combination"," C/T"," TZP","Quinolone",

" GPNX"," STFX"," CIP"," TFLX"," MXF"," LVX"," Sepsis")

f_ROR1<-function(ds,ind_vari){

dat_comp<-na.omit(ds[,c("AE",ind_vari)])

ind_vari1<-ind_vari[1]

if(length(ind_vari)>=2){

for(i in 2:length(ind_vari)){

ind_vari1<-paste(ind_vari1,"+",ind_vari[i],sep="")

}

}

eval(parse(text=paste("logi<-glm(AE~",ind_vari1,",data=ds,family=binomial('logit'))",sep="")))

coe<-summary(logi)$coefficients

resROR<-cbind(round(cbind(exp(coe[-1,1,drop=F]),exp(coe[-1,1]-qnorm(0.975)*

coe[-1,2]),exp(coe[-1,1]+qnorm(0.975)*coe[-1,2]),coe[-1,4]),3))

return(resROR)

}

ROR0<-rbind(rep(NA,4),rep(NA,4),rep(NA,4),f_ROR1(dat_PT,"sex2"),rep(NA,4),f_ROR1(dat_PT,"age2"),rep(NA,4),

f_ROR1(dat_PT,"Cephem"),f_ROR1(dat_PT,"type1"),f_ROR1(dat_PT,"Carbapenem"), f_ROR1(dat_PT,"type2"),

f_ROR1(dat_PT,"type3"),f_ROR1(dat_PT,"type4"),f_ROR1(dat_PT,"beta"),f_ROR1(dat_PT,"type5"),

f_ROR1(dat_PT,"type6"),f_ROR1(dat_PT,"Quinolone"),f_ROR1(dat_PT,"type7"),f_ROR1(dat_PT,"type8"),

f_ROR1(dat_PT,"type9"),f_ROR1(dat_PT,"type10"),f_ROR1(dat_PT,"type11"), f_ROR1(dat_PT,"type12"),

f_ROR1(dat_PT,"indi_pt1a"))

ROR0[ROR0[,1]==0,]<-NA

p<-format(ROR0[,4],nsmall=3)

p[p=="0.000"]<-"<0.001"

p[p=="1.000"]<-">0.999"

ROR1<-cbind(paste(format(ROR0[,1],nsmall=3)," [",gsub(" ","",format(ROR0[,2],

nsmall=3)),"-",gsub(" ","",format(ROR0[,3],nsmall=3)),"]",sep=""),p)

ROR1[rownames(ROR0)=="",]<-""

ROR1[rownames(ROR0)=="type5",]<-"-"

Table1<-cbind(Table1[,2:1],ROR1)

colnames(Table1)<-c("Liver injury","Non-liver injury","ROR [95% CI]","p–value")

#Flowchart

text0<-paste("Total report receiving antibiotics between\nJanuary ",start_y,

" and Semptember ",end_y," (n = ",format(N0,big.mark=","),")",sep="")

text01<-paste("Duplication (n = ",format(N0-N1,big.mark=','),")",sep="")

text1<-paste("n = ",format(N1,big.mark=","),sep="")

text21<-paste("The fourth–generation\n cephalosporin (n = ",format(nrow(subset(dat_PT,

dat_PT$Cephem==1)), big.mark=','),")",sep="")

text22<-paste("Carbapenem (n = ",format(nrow(subset(dat_PT,dat_PT$Carbapenem==1)), big.mark=','),")",sep="")

text23<-paste("β-lactam and β-lactamase\n inhibitor combination\n (n = ",

format(nrow(subset(dat_PT,dat_PT$beta==1)),big.mark=','),")",sep="")

text24<-paste("Quinolone (n = ",format(nrow(subset(dat_PT,dat_PT$Quinolone==1)), big.mark=','),")",sep="")

text31<-paste("FEP (n = ",format(nrow(subset(dat_PT,dat_PT$type1==1)), big.mark=','),")",sep="")

text32<-paste("DOR (n = ",format(nrow(subset(dat_PT,dat_PT$type2==1)), big.mark=','),")",sep="")

text33<-paste("BIPM (n = ",format(nrow(subset(dat_PT,dat_PT$type3==1)), big.mark=','),")",sep="")

text34<-paste("MEM (n = ",format(nrow(subset(dat_PT,dat_PT$type4==1)), big.mark=','),")",sep="")

text35<-paste("C/T (n = ",format(nrow(subset(dat_PT,dat_PT$type5==1)), big.mark=','),")",sep="")

text36<-paste("TZP (n = ",format(nrow(subset(dat_PT,dat_PT$type6==1)), big.mark=','),")",sep="")

text37<-paste("GPNX (n = ",format(nrow(subset(dat_PT,dat_PT$type7==1)), big.mark=','),")",sep="")

text38<-paste("STFX (n = ",format(nrow(subset(dat_PT,dat_PT$type8==1)), big.mark=','),")",sep="")

text39<-paste("CIP (n = ",format(nrow(subset(dat_PT,dat_PT$type9==1)), big.mark=','),")",sep="")

text310<-paste("TFLX (n = ",format(nrow(subset(dat_PT,dat_PT$type10==1)), big.mark=','),")",sep="")

text311<-paste("MXF (n = ",format(nrow(subset(dat_PT,dat_PT$type11==1)), big.mark=','),")",sep="")

text312<-paste("LVF (n = ",format(nrow(subset(dat_PT,dat_PT$type12==1)), big.mark=','),")",sep="")

dev.new(width=4,height=6.5,unit="in")

par(mar=c(0,0,0,0),mgp=c(0,0,0)); par(oma=c(0.1,0.1,0.1,0.1)); par(ps=8)

frsz<-0.13; hposi0<-0.1; dif1<-0.8

plot(c(),xlim=c(0,15),ylim=c(0,12.5),xlab="",ylab="",axes=F)

xt0<-0; yt0<-12.4

text(xt0,yt0,text0,adj=0)

sw0<-strwidth(text0)

sh0<-strheight(text0)

xt00<-xt0-frsz

rect(xt00,yt0-sh0/2-frsz,xt0+sw0+frsz,yt0+sh0/2+frsz)

xt1<-0; yt1<-yt0-frsz*2-dif1*2

text(xt1,yt1,text1,adj=0)

sw1<-strwidth(text1)

sh1<-strheight(text1)

xt1l<-xt1-frsz

rect(xt1l,yt1-sh1/2-frsz,xt1+sw1+frsz,yt1+sh1/2+frsz)

sw<-strwidth(text22)+dif1

sh<-strheight(text22)

f1<-function(xt0,yt0,text0,sw,sh){

text(xt0,yt0,text0,adj=0)

rect(xt0-frsz,yt0-sh/2-frsz,xt0+sw+frsz,yt0+sh/2+frsz)

}

xt2<-xt1+hposi0; yt2<-yt1

f1(xt2+dif1*2,yt0-sh/2-frsz-dif1*1,text01,strwidth(text22)+dif1,strheight(text22))

f1(xt2+dif1*2,yt2-sh/2-frsz-dif1*1,text21,strwidth(text22)+dif1,strheight(text22)*2.2)

f1(xt2+dif1*2,yt2-sh/2-frsz-dif1*2,text22,strwidth(text22)+dif1,strheight(text22))

f1(xt2+dif1*2,yt2-sh/2-frsz-dif1*5,text23,strwidth(text22)+dif1,strheight(text22)*3.3)

f1(xt2+dif1*2,yt2-sh/2-frsz-dif1*7,text24,strwidth(text22)+dif1,strheight(text22))

yt3<-yt2; xt3<-9

f1(xt3+dif1*2,yt3-sh/2-frsz-dif1*1,text31,strwidth(text33)+dif1,strheight(text33))

f1(xt3+dif1*2,yt3-sh/2-frsz-dif1*2,text32,strwidth(text33)+dif1,strheight(text33))

f1(xt3+dif1*2,yt3-sh/2-frsz-dif1*3,text33,strwidth(text33)+dif1,strheight(text33))

f1(xt3+dif1*2,yt3-sh/2-frsz-dif1*4,text34,strwidth(text33)+dif1,strheight(text33))

f1(xt3+dif1*2,yt3-sh/2-frsz-dif1*5,text35,strwidth(text33)+dif1,strheight(text33))

f1(xt3+dif1*2,yt3-sh/2-frsz-dif1*6,text36,strwidth(text33)+dif1,strheight(text33))

f1(xt3+dif1*2,yt3-sh/2-frsz-dif1*7,text37,strwidth(text33)+dif1,strheight(text33))

f1(xt3+dif1*2,yt3-sh/2-frsz-dif1*8,text38,strwidth(text33)+dif1,strheight(text33))

f1(xt3+dif1*2,yt3-sh/2-frsz-dif1*9,text39,strwidth(text33)+dif1,strheight(text33))

f1(xt3+dif1*2,yt3-sh/2-frsz-dif1*10,text310,strwidth(text33)+dif1,strheight(text33))

f1(xt3+dif1*2,yt3-sh/2-frsz-dif1*11,text311,strwidth(text33)+dif1,strheight(text33))

f1(xt3+dif1*2,yt3-sh/2-frsz-dif1*12,text312,strwidth(text33)+dif1,strheight(text33))

lines(c(xt0+dif1,xt0+dif1),c(yt0-sh0/2-frsz,yt1+sh/2+frsz))

lines(c(xt1+dif1,xt1+dif1),c(yt1-sh/2-frsz,yt2-sh/2-frsz-dif1*7))

lines(c(xt1+dif1,xt2+dif1*2-frsz),c(yt0-sh/2-frsz-dif1*1,yt0-sh/2-frsz-dif1*1))

lines(c(xt1+dif1,xt2+dif1*2-frsz),c(yt2-sh/2-frsz-dif1*1,yt2-sh/2-frsz-dif1*1))

lines(c(xt1+dif1,xt2+dif1*2-frsz),c(yt2-sh/2-frsz-dif1*2,yt2-sh/2-frsz-dif1*2))

lines(c(xt1+dif1,xt2+dif1*2-frsz),c(yt2-sh/2-frsz-dif1*5,yt2-sh/2-frsz-dif1*5))

lines(c(xt1+dif1,xt2+dif1*2-frsz),c(yt2-sh/2-frsz-dif1*7,yt2-sh/2-frsz-dif1*7))

lines(c(xt2+dif1*2+sw+frsz,xt3+dif1*2-frsz),c(yt2-sh/2-frsz-dif1*1,yt2-sh/2-frsz-dif1*1))

lines(c(xt2+dif1*2+sw+frsz,xt3+dif1*2-frsz),c(yt2-sh/2-frsz-dif1*2,yt2-sh/2-frsz-dif1*2))

lines(c(xt2+dif1*2+sw+frsz,xt3+dif1*2-frsz),c(yt2-sh/2-frsz-dif1*5,yt2-sh/2-frsz-dif1*5))

lines(c(xt2+dif1*2+sw+frsz,xt3+dif1*2-frsz),c(yt2-sh/2-frsz-dif1*7,yt2-sh/2-frsz-dif1*7))

lines(c((xt2+dif1*2+sw+frsz+xt3+dif1*2-frsz)/2,(xt2+dif1*2+sw+frsz+xt3+

dif1*2-frsz)/2),c(yt2-sh/2-frsz-dif1*2,yt2-sh/2-frsz-dif1*4))

lines(c((xt2+dif1*2+sw+frsz+xt3+dif1*2-frsz)/2,(xt2+dif1*2+sw+frsz+xt3+

dif1*2-frsz)/2),c(yt2-sh/2-frsz-dif1*5,yt2-sh/2-frsz-dif1*6))

lines(c((xt2+dif1*2+sw+frsz+xt3+dif1*2-frsz)/2,(xt2+dif1*2+sw+frsz+xt3+

dif1*2-frsz)/2),c(yt2-sh/2-frsz-dif1*7,yt2-sh/2-frsz-dif1*12))

lines(c((xt2+dif1*2+sw+frsz+xt3+dif1*2-frsz)/2,xt3+dif1*2-frsz), c(yt2-sh/2-frsz-dif1*3,yt2-sh/2-frsz-dif1*3))

lines(c((xt2+dif1*2+sw+frsz+xt3+dif1*2-frsz)/2,xt3+dif1*2-frsz),c(yt2-sh/2-frsz-dif1*4,yt2-sh/2-frsz-dif1*4))

lines(c((xt2+dif1*2+sw+frsz+xt3+dif1*2-frsz)/2,xt3+dif1*2-frsz),c(yt2-sh/2-frsz-dif1*6,yt2-sh/2-frsz-dif1*6))

lines(c((xt2+dif1*2+sw+frsz+xt3+dif1*2-frsz)/2,xt3+dif1*2-frsz),c(yt2-sh/2-frsz-dif1*8,yt2-sh/2-frsz-dif1*8))

lines(c((xt2+dif1*2+sw+frsz+xt3+dif1*2-frsz)/2,xt3+dif1*2-frsz),c(yt2-sh/2-frsz-dif1*9,yt2-sh/2-frsz-dif1*9))

lines(c((xt2+dif1*2+sw+frsz+xt3+dif1*2-frsz)/2,xt3+dif1*2-frsz),c(yt2-sh/2-frsz-dif1*10,yt2-sh/2-frsz-dif1*10))

lines(c((xt2+dif1*2+sw+frsz+xt3+dif1*2-frsz)/2,xt3+dif1*2-frsz), c(yt2-sh/2-frsz-dif1*11,yt2-sh/2-frsz-dif1*11))

lines(c((xt2+dif1*2+sw+frsz+xt3+dif1*2-frsz)/2,xt3+dif1*2-frsz), c(yt2-sh/2-frsz-dif1*12,yt2-sh/2-frsz-dif1*12))

#Table 1

Table1

#Save rawdata as csv file in "C:\FAERS"

write.csv(rawdata,"C:\\FAERS\\rawdata.csv")Supplementary 8. Common Terminology Criteria for Adverse Events ver. 5.0: elevation of alanine aminotransferase

|  | Grade 1 | Grade 2 | Grade 3 | Grade 4 |
| --- | --- | --- | --- | --- |
| Male | Baseline ≤42 U/L:  >42-126 U/L  Baseline >42 U/L:  1.5-3.0×baseline | Baseline ≤42 U/L:  >126-210 U/L  Baseline >42 U/L:  >3.0-5.0×baseline | Baseline ≤42 U/L:  >210-840 U/L  Baseline >42 U/L:  >5.0-20.0×baseline | Baseline ≤42 U/L:  >840 U/L  Baseline >42 U/L:  >20.0×baseline |
| Female | Baseline ≤23 U/L:  >23-69 U/L  Baseline >23 U/L:  1.5-3.0×baseline | Baseline ≤23 U/L:  >69-115 U/L  Baseline >23 U/L:  >3.0-5.0×baseline | Baseline ≤23 U/L:  >115-460 U/L  Baseline >23 U/L:  >5.0-20.0×baseline | Baseline ≤23 U/L:  >460 U/L  Baseline >23 U/L:  >20.0×baseline |
